# The adsorption of drugs on nanoplastics has severe biological impact

**DOI:** 10.1038/s41598-024-75785-4

**Published:** 2024-10-28

**Authors:** Leonard Dick, Patrick R. Batista, Paul Zaby, Gabriele Manhart, Verena Kopatz, Lukas Kogler, Verena Pichler, Florian Grebien, Vince Bakos, Benedek G. Plósz, Nikola Zlatkov Kolev, Lukas Kenner, Barbara Kirchner, Oldamur Hollóczki

**Affiliations:** 1 Mulliken Center for Theoretical Chemistry, University of Bonn, Beringstr. 4 6, 53115 Bonn, Germany; 2 Department of Colloid Chemistry, Max Planck Institute of Colloids and Interfaces, Am Mühlenberg 1, 14476 Potsdam, Germany; 3 Institute of Chemistry, University of Campinas, Monteiro Lobato, 270, Cidade Universitária, 13083-862 Campinas, São Paulo Brazil; 4 Medical Biochemistry, Department for Biological Sciences and Pathobiology, University of Veterinary Medicine Vienna, 1210 Vienna, Austria; 5 CeMM Research Center for Molecular Medicine of the Austrian Academy of Sciences, 1090 Vienna, Austria; 6 Department for Experimental and Laboratory Animal Pathology, Medical University of Vienna, Clinical Institute of Pathology, 1090 Vienna, Austria; 7 Center for Biomarker Research in Medicine (CBmed GmBH), microOne, 8010 Graz, Austria; 8 Department for Radiation Oncology, Medical University of Vienna, 1210 Vienna, Austria; 9 Comprehensive Cancer Center Vienna, Medical University of Vienna, 1090 Vienna, Austria; 10 Department of Biomedical Imaging and Image-guided Therapy, Medical University of Vienna, 1090 Vienna, Austria; 11 Division of Pharmaceutical Chemistry, University of Vienna, 1090 Vienna, Austria; 12 St. Anna Children’s Cancer Research Institute (CCRI), 1090 Vienna, Austria; 13 Department of Chemical Engineering, University of Bath, Claverton Down, Bath, BA2 7AY UK; 14 Department of Applied Biotechnology and Food Science, Budapest University of Technology and Economics, Műegyetem rkp. 3, 1111 Budapest, Hungary; 15 Department of Molecular Biology, Umeå University, Umeå, Sweden; 16 Christian Doppler Laboratory for Applied Metabolomics, Medical University of Vienna, 1090 Vienna, Austria; 17 Unit of Laboratory Animal Pathology, University of Veterinary Medicine Vienna, 1210 Vienna, Austria; 18 Department of Physical Chemistry, Faculty of Science and Technology, University of Debrecen, Egyetem tér 1, 4032 Debrecen, Hungary

**Keywords:** Nanotoxicology, Nanoscale biophysics, Medical research

## Abstract

Micro- and nanoplastics can interact with various biologically active compounds forming aggregates of which the effects have yet to be understood. To this end, it is vital to characterize these aggregates of key compounds and micro- and nanoplastics. In this study, we examined the adsorption of the antibiotic tetracycline on four different nanoplastics, made of polyethylene (PE), polypropylene (PP), polystyrene (PS), and nylon 6,6 (N66) through chemical computation. Two separate approaches were employed to generate relevant conformations of the tetracycline-plastic complexes. In the first approach, we folded the plastic particle from individual polymer chains in the presence of the drug through multiple separate simulated annealing setups. In the second, more biased, approach, the neat plastic was pre-folded through simulated annealing, and the drug was placed at its surface in multiple orientations. The former approach was clearly superior to the other, obtaining lower energy conformations even with the antibiotic buried inside the plastic particle. Quantum chemical calculations on the structures revealed that the adsorption energies show a trend of decreasing affinity to the drug in the order of N66> PS> PP> PE. In vitro experiments on tetracycline-sensitive cell lines demonstrated that, in qualitative agreement with the calculations, the biological activity of tetracycline drops significantly in the presence of PS particles. Preliminary molecular dynamics simulations on two selected aggregates with each plastic served as first stability test of the aggregates under influence of temperature and in water. We found that all the selected cases persisted in water indicating that the aggregates may be stable also in more realistic environments. In summary, our data show that the interaction of micro- and nanoplastics with drugs can alter drug absorption, facilitate drug transport to new locations, and increase local antibiotic concentrations, potentially attenuating antibiotic effect and at the same time promoting antibiotic resistance.

## Introduction

Through the degradation of plastics, particles with a wide variety of sizes, shapes, and compositions are formed^1–5^. Over the past few decades, it has become evident that micro- and nanometer-sized fragments of these pollutants (micro- and nanoplastic particles, MNPs) are present in our environment^1,2,6,7^. Beyond the fact that these artificial materials enter the human body^8^, organs^9–14^, or even individual cells^15^ of living organisms, their physiological and environmental effects are not yet fully understood^16–18^. Numerous studies have focused on understanding the consequences of absorbing MNPs, and reached alarming conclusions^5,15,19–25^. While it is clear that decreasing the MNP intake is necessary to mitigate the risks, more research is needed to identify the particular kinds of MNPs that pose a health and environmental danger.

When assessing the possible effects of MNPs, it is crucial to consider their interactions with other compounds in natural and artificial environments. These materials can adsorb a wide variety of substances and, through a “Trojan horse” effect, deliver them into living organisms. Compounds called endocrine disruptors (EDCs)^26^ that enter life forms through this mechanism can trigger severe toxic effects on the hormonal level, which is suspected to be linked to the increased incidence of cancer cases worldwide^5^, but also male infertility^27^.

The interaction of MNPs with medicinal drugs can also have severe consequences. First, through the aforementioned “Trojan horse” effect, residual drugs in waste water^28^ can be shuttled into the body of humans and animals, resulting in unintended physiological changes^29–33^. Second, it is conceivable that when MNPs are present in the digestive tract of the patient while undergoing medical treatment through oral delivery, interactions between the drug and the MNP particles may slow down, or even hinder the absorption of the drug into the blood stream, thereby altering the pharmacokinetics of the medication. This hypothesis must be rigorously tested with the ever-increasing particle load in humans due to the rising concentration of MNPs in the environment.

However, when considering MNP-drug interactions, antimicrobial agents deserve special attention, as the possible negative effects can be significantly exacerbated by these compounds. The presence of antibiotic drugs in the environment is of great concern even without their interplay with MNPs^34^. Depending on the exposure level, bacteria exposed to these compounds may exhibit intrinsic, adaptive and acquired resistance (i.e. positive selection above minimal selective concentration, MSC). Still, even much below MSC, the persistence of resistance genes may increase^35^. Due to this evolutionary pressure, and the continuous release of antimicrobial compounds by their anthropogenic use and misuse, the abundance of resistance genes has been observed to increase in the environment^36^. The emergence and spread of resistant strains is highly alarming, since common infectious diseases have become a global threat. The findings above prompted the definition of new strategies and policies for assessing drugs’ environmental and human health risks^37^, including the potential contribution to antimicrobial resistance when selecting the compound’s Predicted No Effect Concentration. As the most common form of bacterial life^38^, biofilms are bacterial populations embedded in self-produced heterogeneous matrices where diffusion, sorption^39^ and biotransformation^40^ of antimicrobial chemicals may occur. MNPs provide a surface for microorganisms to colonize, serving as a vector for transportation and spread^41^. The local increase in drug concentration induced by the MNP leads to the exposure of bacteria to higher quantities of the drug than in the absence of the plastic, increasing the chances for resistance to develop. Furthermore, both microplastics and biofilms seem to facilitate the transfer of resistance genes between the microbes^42,43^. However, a deeper and more structured understanding of analyte-MNP, analyte-biofilm, and biofilm-MNP interactions may be required to bridge knowledge gaps regarding the development and spread of antimicrobial resistance in complex environmental matrices.

**Fig. 1:**
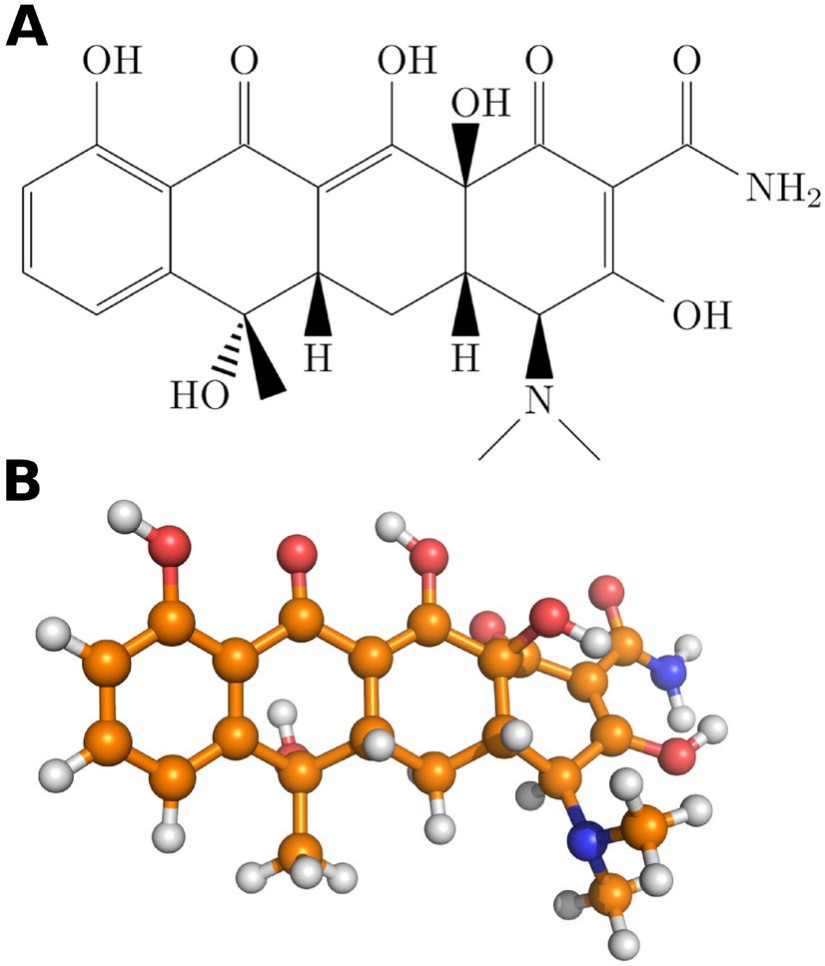
Lewis structure (**A**) and ball-and-stick representation (**B**) of the antibiotic tetracycline (carbon: orange; hydrogen: white; oxygen: red; nitrogen: blue).

Thus, beyond being potentially toxic themselves, MNPs can also offer a platform, at which resistant strains of bacteria can be produced. If a post-antibiotic era is to be avoided, all possible actions need to be taken to explore and eliminate the sources of resistance, and the connections with plastic waste need to be unveiled. Understanding the key features of drug adsorption on MNPs is essential in this endeavour. Several studies have been published throughout the last decade that focused on the interaction of MNPs and antibiotics^44–49^, including a broad-spectrum antibiotic, tetracycline (TC, see Fig. 1). The importance of this particular drug is underscored by the fact that it is on the WHO’s list of essential medicines, despite its significantly eroded versatility. Further loss of its efficacy will have a devastating effect on the treatment of some severe infections, including malaria and syphilis.

TC binds spontaneously to polyethylene (PE), polypropylene (PP), polystyrene (PS), and polyvinyl chloride (PVC)^44,49^, following a multilayer adsorption mechanism^49^, changing the morphology of the MNP particle^48^. The interactions that drive this adsorption include polar and $\pi - \pi$ interactions, making PVC^48^ and PS the strongest adsorbents among the investigated MNPs, followed by PP and finally PE^45^. Weathering introduces polar functional groups to the plastics, such as carbonyl groups, which enhance adsorption^47,50^. Organic and inorganic substances can also significantly affect sorption^44–46,50^, therefore the particular matrix, in which the binding occurs, has to be considered in the experimental setups and their interpretation.

**Fig. 2:**
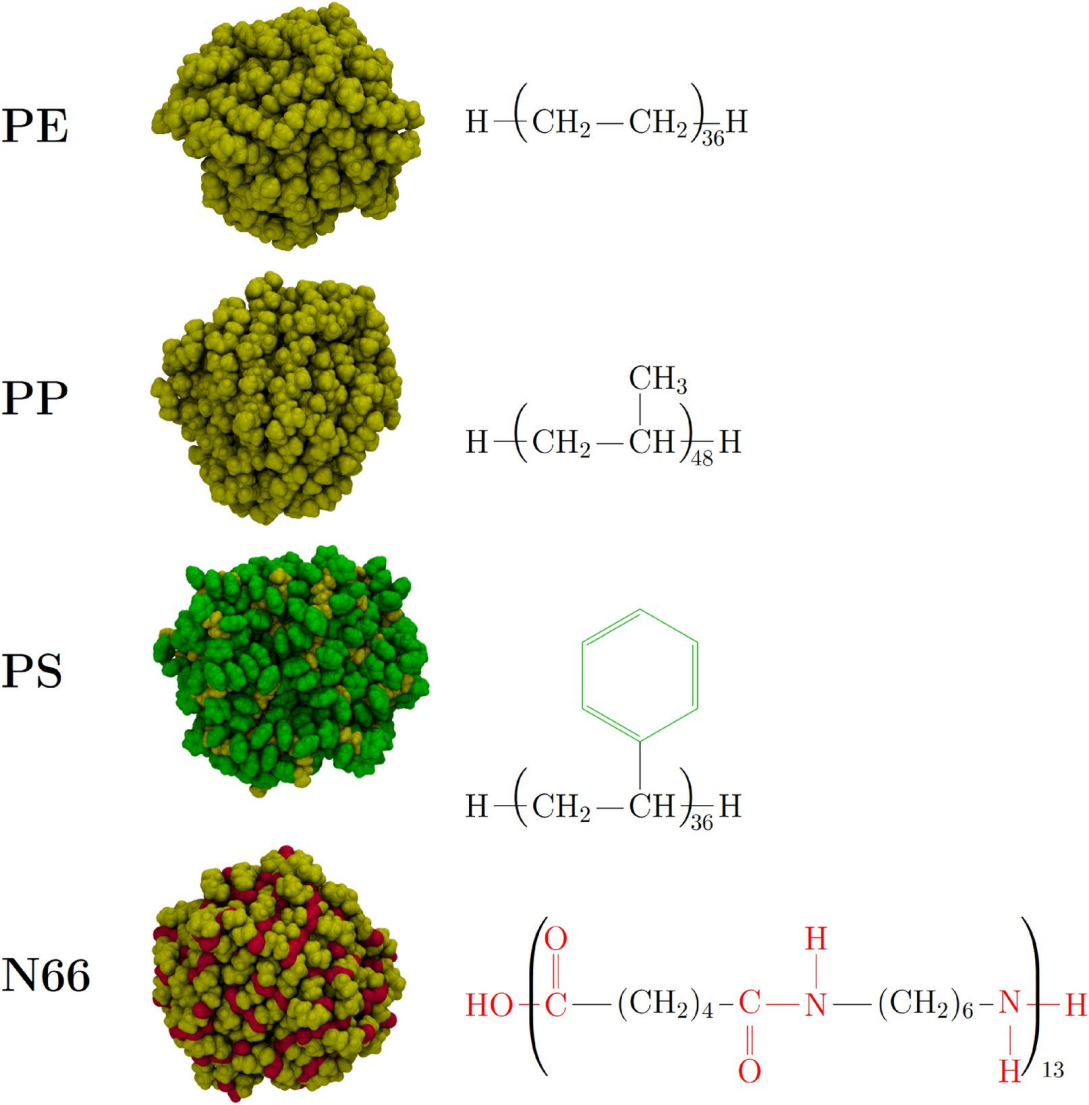
Ball-and-stick structures and Lewis formulae of the four investigated NP particles; characteristic functional groups of polystyrene (PS, phenyl groups) and nylon (N66, amide units) are highlighted in green and red, respectively. The composition and number of polymer chains use of each NP are given in Supplementary Table S1.

In the present article, we use a theoretical chemical and molecular biological approach to characterize how TC interacts with a variety of nanoplastics, and to observe how the biological activity of the drug is altered by the presence of these plastic pollutants. Since this antibiotic interacts weaker with MNPs than many other antibiotics (ciprofloxacin, amoxicillin, sulfadiazine, trimethoprin)^44^, investigating TC should be more challenging for modeling than the others. For the same reason, any effects found for TC in the experiments should be stronger with the other drugs, providing a careful underestimation for the general biolocial impact of MNP-drug interactions. We chose four kinds of pristine plastics for our study, including the plastics produced in the most significant quantities such as polyethylene (PE), polypropylene (PP), and polystyrene (PS), which are also the most prevalent in the environment, and nylon 6,6 (N66), which has been shown to have severe effects on biomolecules (see Fig. 2)^51,52^. Note, in the following, we use only NP as an abbreviation for the micro- and nanoplastic (MNP) because the size of our investigated subjects does not exceed the nanoscale.

## Methods

### Theoretical chemical calculations

Exploring the conformational space of macromolecular systems is a difficult task, and beyond a certain size only stochastic methods are available. One of these methods is simulated annealing (SA)^53–58^, which relies on a temperature program, heating up the system to an often unphysically high temperature, and then gradually cooling it down. The high kinetic energy of the atoms allows the facile exploration of the conformations of the system, while the gradual cooling depopulates the higher energy structures. This approach can find the global minimum of the potential energy surface of any system, if the rate of cooling is infinitely low; in any other (i.e. realistic) cases it may produce energetically higher lying minima. Thus, a careful design of the simulated annealing setup is necessary, and it is highly advantageous to perform several parallel SA runs that produce multiple conformations^58^.

In order to obtain several conformations of the drug molecule TC with the nanoplastic, we adapted a workflow from our earlier established^51^ simulated annealing protocol. First, the polymer chains that the plastic particle is to be composed of were placed in a periodic box. The box size was chosen to be large enough to avoid close self-interactions between the opposite ends of the same polymer chain, but small enough to prevent the individual chains from floating apart, e.g. 150 Å. On this system, a molecular dynamics run of 20 ns was performed in the canonical ensemble with the temperature gradually decreasing from 1200 to 200 K. The radius of gyration of the particle and the mobility of the chains within were monitored through the simulation, showing the phase transition temperatures at which first, the polymer chains condense into a single cluster (defining T$_{start}$), and second, their mobility becomes so limited that they do not explore their conformational space anymore (defining T$_{end}$). These two temperatures were taken as the two extreme points for the subsequent simulated annealing runs. Then, 50 separate starting conformations were generated, and on each of these a simulated annealing run was performed in the canonical ensemble for 20 ns, cooling gradually from the obtained T$_{start}$ to the T$_{end}$ temperature.

Next, we built the TC@NP system in two separate ways. In one approach, we carried out a complete refolding of NP in the presence of the TC through the same temperature program designed for the neat plastic, i.e., SA was applied to the TC@NP systems as well. We again performed these simulations on 50 different starting structures, so that we received an array of conformations at the end. In the other approach, we placed the TC on the pre-folded NP surface that was found as the lowest energy structure of the simulated annealing runs of the pure plastic. We term this FP (for free particle) approach. The TC was attached to the surface of the plastic in various systematically chosen orientations. A 1 ns molecular dynamics simulations run at 200 K followed to adjust the position and orientation of the drug. These two processes led to a total of 104 aggregates for each NP.

To characterize the binding modes of the chosen drug TC to the various plastics, each of the obtained multiple conformations underwent a geometry optimization based on the OPLS-AA^59,60^ force field. Subsequently, we carried out semi-empirical quantum chemical calculations with implicit aqueous solvation, using the xtb program^61^, and the GFN2-xTB method^62^. The structures were all fully geometry optimized. The binding modes and sorption energies discussed in the paper were obtained from these quantum chemical calculations. The methodology is described in the Supporting Information (see Supplementary Fig. S2).

To study the temperature influence and solvation behavior of the TC@NP aggregates, we performed two molecular dynamics simulations for each type of plastic, with 32000 water molecules explicitly modeled using the Lammps^63^ program package with the OPLS-AA force field for the NP and TC, and the SPC/E^64^ water model. The bond and angle terms of water were constrained throughout the simulations using the SHAKE^65^ algorithm to allow for a time step of 1 fs. Pairwise interactions were computed up to a cut off distance of 10 Å. Coulombic interactions for larger distances were computed using the paricle-particle particle-mesh method. The equilibration process was initiated with a minimization with the convergence criteria set to 10$^{-4}$ kcal mol$^{-1}$ for the energy and 10$^{-4}$ kcal mol$^{-1}$ Å$^{-1}$ for the force term. After convergence, a simulation in a microcanonical ensemble was performed to further remove hotspots in the initial configuration over 50 ps, while explicitely rescaling the temperature of the system at 500 K. The temperature was then adjusted in a short NVT ensemble to 293.15 K via a temperature ramp over 100 ps. After the thermal adjustment, the setup was changed to an NpT ensemble to adjust the density over 5 ns. The box volume was averaged over the last 3 ns before adjusting the cell vector to the calculated value. The final cell vectors are listed in the Supporting Information (see Supplementary Table S2). For the final equilibration phase of 0.7 ns and the subsequent production run, the NVT ensemble was used. In the production run of 50 ns, every 10,000-th time step was saved to the trajectory file and later used for analysis. The processing of the trajectories and all of the analyses presented in this paper were carried out using the TRAVIS program suite^66,67^.

### Molecular biology experiments

**Table 1:** Particle characterization of the studied plastic particles. The sizes are given as listed by the commercial supplier. Micro- and nanoplastic particles were additionally analyzed for $\zeta$ potential, size distribution and polydispersity index (PDI) (1 mg particles in 1 mM KCl).

	Size (supplier)/$\upmu$m	$\zeta$ potential	Size (measured)/nm	PDI (measured)
PET	−	−34.30	1090	0.26
PE	0.2–9.9	−34.72	n.d.	n.d.
PS 1 $\upmu$m	1.11	–71.75	1158	0.05
PS 0.25 $\upmu$m	0.252	−64.51	258.3	0.01

Microplastic particles were either purchased commercially—polystyrene (PS, microParticles GmbH, Germany; 250 nm or 1.1 $\upmu$m mean particle size, 5% or 10% w/vol in aqueous solution), polyethylene (PE, Cospheric LLC, United States; size range 0.2–9.9 $\upmu$m, dissolved at 100 mg/ml in 1% Tween20)—or produced in the lab—polyethylene terephthalate (PET, mean size range of 1.9 ± 0.3 $\upmu$m, dissolved at 630 $\upmu$g/ml in 0.5% Tween 20) using a variation of the protocol of Rodríguez-Hernández et al.^68^ PS particle characterization was performed measuring $\zeta$ potential, size and polydispersity index (PDI) on a Zetasizer Pro device (Malvern Pananalytical, Malvern, United Kingdom). Data analysis was performed using ZS Xplorer software. $\zeta$ potential, size and PDI of 1 mg particles were measured in 1 mM KCl solution (see Table 1). DLS measurements for PE particle were not possible due to the high particles size range. For scanning electron microscopy (SEM) PET particles were dried and mounted on aluminum stubs with conductive tape and Au sputter-coated for 120 s at 50 mA using a sputter-coating device Q150R ES from Quorum Technologies Ltd. (East Grinstead, UK). The SEM images (see Supplementary Fig. S19) were measured using a JEOL JSM-6510 scanning electron microscope from JEOL GmbH (Eching/Munich, Germany). SEM images of commercially supplied particles are provided by the company on request via https://www.microparticles-shop.de/, or https://www.cospheric.com/. A platinum ATR alpha from Bruker (Billerica, USA) with its software OPUS 7.5, was used for FTIR-measurements of all particles (see Supplementary Fig. S20). Light microscopy was performed on an Invitrogen EVOS FL imaging system (see Supplementary Fig. S19). Both mouse (Cebpa$^{\mathrm {p30/p30}}$ shCebpa) and human (MV4:11 shRen.713) cell lines were cultured as described previously (Schmidt et al.^69^ and Skucha et al.^70^, respectively). In these cells, expression of the shRNA and the fluorescent reporter protein is under the regulation of a tetracycline-controlled promoter (Tet-On)^71^. Microplastic samples were pre-incubated in fetal bovine serum (FBS) for 20 minutes at room temperature, before incubation at given concentrations with tetracycline (CycloPel 300mg capsules, Pelpharma, Cat# 136100, diluted to 16 $\upmu$g/mL in cell culture medium) at 37 $^\circ$C for 1 h. Tetracycline ± microplastic was added to the cells for 45 h before measuring the induction of the reporter on the IQue3 (Sartorius). Data were analyzed using Forecyte 10.0 and FlowJo 10.4.2 and statistical analysis was done with Excel (2308) and GraphPad Prism (6.01). To evaluate the statistical significance of the differences in adsorption rates among various experimental conditions, we conducted a One-Way Analysis of Variance (ANOVA). This method was selected as it allows for comparison of means across multiple groups, providing insights into whether any significant differences exist.

## Results

### Conformational analysis

The obtained structures of the neat NPs resemble the known features of bulk plastics of the same material, indicating that simulated annealing works reasonably well for these systems. For instance, the most stable N66 particle exhibits a clear structure, in which the amide groups line up to form long chains through hydrogen bonding (red in Fig. 2) that repeat across the roughly spherical plastic, analogously to the crystal structure of this highly crystalline polymer. The folding of multiple chains of these hydrogen bonds into a sphere produces a complex network of mesoscopic structures, as shown in Fig. 2. The three remaining plastics—in which the chains are held together only by weak intermolecular interactions—are often amorphous, also represented in the hereby obtained geometries, lacking any clearly recognizable features. The detailed structure of these particles has been described elsewhere^51,52^.

The surface structure of the particle determines its solvation and the interactions with any molecule that is adsorbed on its surface. Both PE and PP consist solely of non-polar functional groups, therefore their surface lacks hydrogen bonding sites. However, the surface of PP should be rougher due to the presence of the methyl groups that hinder the close alignment of the polymer chains. The PS particle exhibits a significant portion of its phenyl functionalities at the surface, pointing away from the rest of the plastic (Fig. 2). The resulting surface contains many cavities and voids, which may affect adsorption processes. N66, on the other hand, has several polar amide groups on its surface, which can form multiple hydrogen bonds with either the aqueous solvent, or the molecule to be adsorbed. Due to these differences in the structure of the various MNPs investigated here, the tetracycline molecule is expected to behave differently when interacting with each particular NP.

Employing the two separate strategies described in the Methods section and the Supporting Information, we produced 104 conformations for each plastic. Simulated annealing is a conformational search approach, and its aim is not to generate a thermodynamic ensemble; hence, strictly speaking, only the lowest energy structures are thermodynamically meaningful. Yet, since it is a stochastic algorithm, the geometries produced can be considered to be a random sample from the conformational space. Similarly, we distributed the TC in the FP approach over the whole NP, producing random orientations (see Supplementary Fig. S1). Therefore, the geometries obtained can be seen as an extension of that sample. Thus, comparing the resulting structures in terms of geometry and energy can lead to a more detailed understanding of TC-NP interactions and allow for a more sophisticated approach to produce the present macromolecular clusters.

It must be stressed here that the inherent features of the SA and FP methods determine some differences in the meaning of the data. First, the plastic is refolded in the SA approach, the relative energies of the structures produced may result from the imperfect alignment of the polymer chains. On the other hand, in the FP method, an already reasonable plastic structure is selected. Therefore, the energy differences are all related solely to the plastic-TC interaction. However, shown below, it is essential to select the proper neat plastic structure for FP if the energies are to be compared directly to those obtained from SA. Second, the refolding allows the plastic to better adjust to the molecule it should interact with, and therefore the ideal modes of plastic-TC interplay can be tracked. In contrast, in FP, only a limited adjustment of the surface to the drug can be expected. Third, somewhat related to the previous point, if the drug should penetrate the plastic particle, i.e., diffuse in between the chains, FP will not be able to recognize the full extent of the complex adsorption behavior that is the focus of our study.

**Fig. 3:**
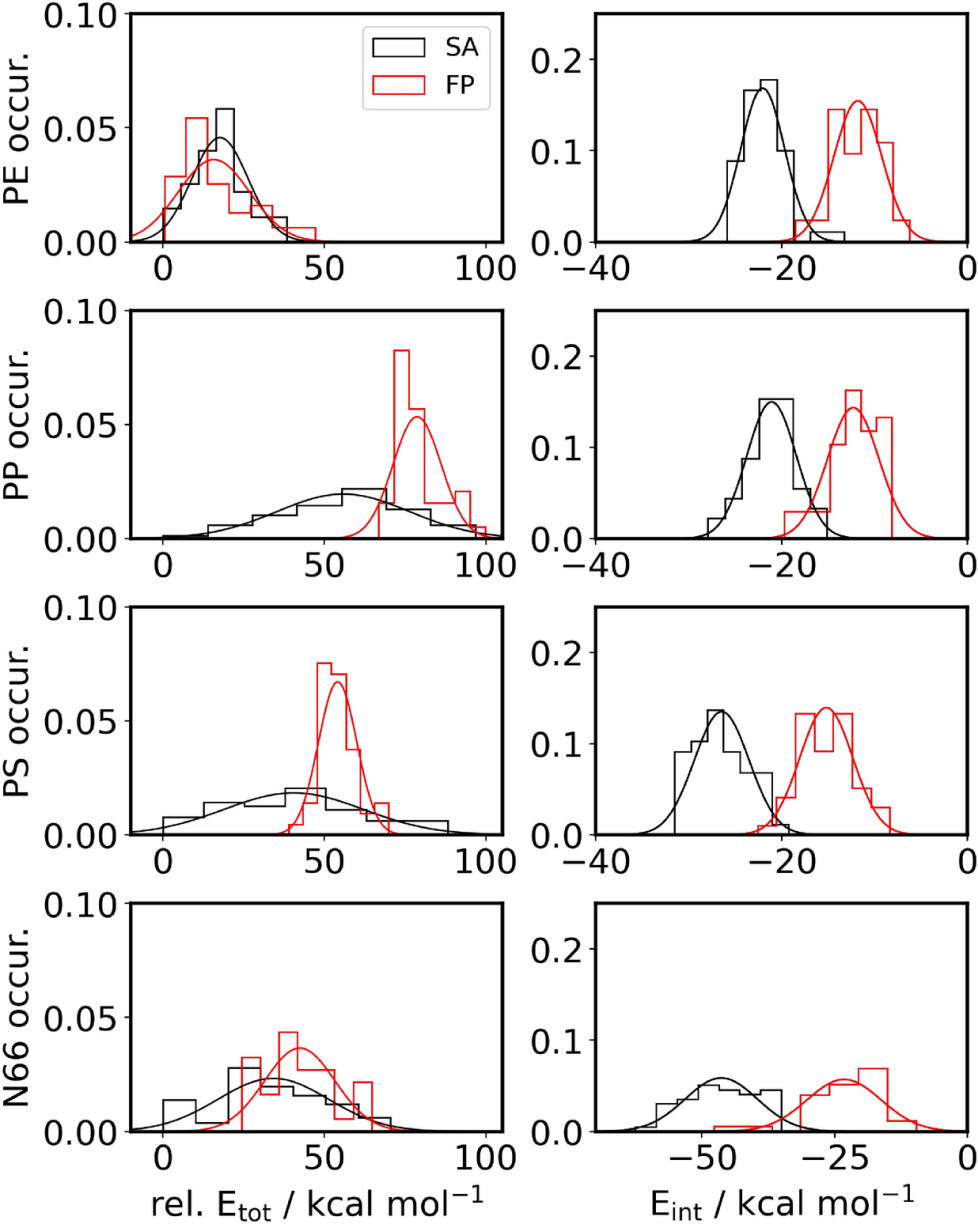
The distribution of relative total energies referenced to minimum value (left) and the interaction energy for the different NP with TC (right) are presented as histograms with bins of 7 chosen according to Sturge’s rule. The normal distribution plots are only to guide the eye.

**Table 2:** Relative total energy in kcal/mol: Collection of mean, standard deviation ($\sigma$), skewness (skew), kurtosis (kurt), x-median (xMed), lowest x-value (xlow) and highest x-value (xhi) for histogram and bootstrapped histogram. SA: simulated annealing approach; SA$^{B}$: SA bootstrapped; FP: fixed particle approach; FP$^{B}$: fixed particle bootstrapped.

	PE				PP			
	SA	SA$^B$	FP	FP$^B$	SA	SA$^B$	FP	FP$^B$
Mean	18	18	16	16	56	56	79	79
$\sigma$	9	1	11	2	20	3	7	1
95 % conf.	3	–	3	–	6	–	2	–
skew	0.32	0.03	1.00	0.16	−0.47	−0.06	1.09	0.16
kurt	−0.16	−0.02	0.26	0.08	−0.23	−0.02	0.46	0.07
xMed	17	18	12	16	60	56	77	79
xlow	0	13	0	10	0	45	67	75
xhi	38	22	47	24	97	66	100	85

	PS				N66			
	SA	SA$^B$	FP	FP$^B$	SA	SA$^B$	FP	FP$^B$
Mean	40	40	54	54	34	34	42	42
$\sigma$	22	3	6	1	17	2	11	2
95 % conf.	6	–	2	–	5	–	4	–
skew	0.20	0.04	0.46	0.00	−0.07	−0.02	0.29	0.01
kurt	−0.65	−0.04	0.88	−0.04	−0.52	−0.03	−0.56	−0.04
xMed	41	40	54	54	31	34	42	42
xlow	0	30	39	51	0	25	24	35
xhi	88	51	70	58	70	42	65	49

The relative total energies of the structures vary considerably (Table 2 and left side of Fig. 3), in the range of up to 100 kcal mol$^{-1}$, which underscores the need for multiple folding attempts to ensure that higher energy conformations and the related erroneous conclusions regarding structures and energies can be avoided. As expected, the FP data (shown in red in Fig. 3) scatters less, as these conformer aggregates differ exclusively in their surface structure. This finding, together with the higher energy values of xlow in Table 2 shows it is inferior to the SA approach. For PE the scatter is the lowest and SA as well FP exhibit similar distributions.

The scatter in SA is greatest in case of PP and PS, as shown by the standard deviation ($\sigma$), the x-median (xMed) and highest x-value (xhi) numbers in Table 2. Note the bootstrapping (SA$^B$ values) of this data reduces the standard deviation to about 3 kcal/mol. Further note, the bootstrapping does not find lower energy values but works within the range of the original data, which is why xlow never reaches 0. The two plastics (PP and PS) are, presumably, due to the functional groups branching off the backbone, apparently more complicated to fold. In other words, the roughness of the chains should produce higher barriers between the minima, which hinders the rearrangement from a high-energy structure into a more stable one. This feature makes simulated annealing more likely to get trapped in a higher energy structure, making SA much more sensitive to the particular setup of the procedure. Thus, the refolding of the TC@NP complexes by the SA approach yields a higher portion of thermodynamically unstable or metastable structures than in the case of the other two plastics (cf. $\sigma$, xMed and xhi values in Table 2).

**Fig. 4:**
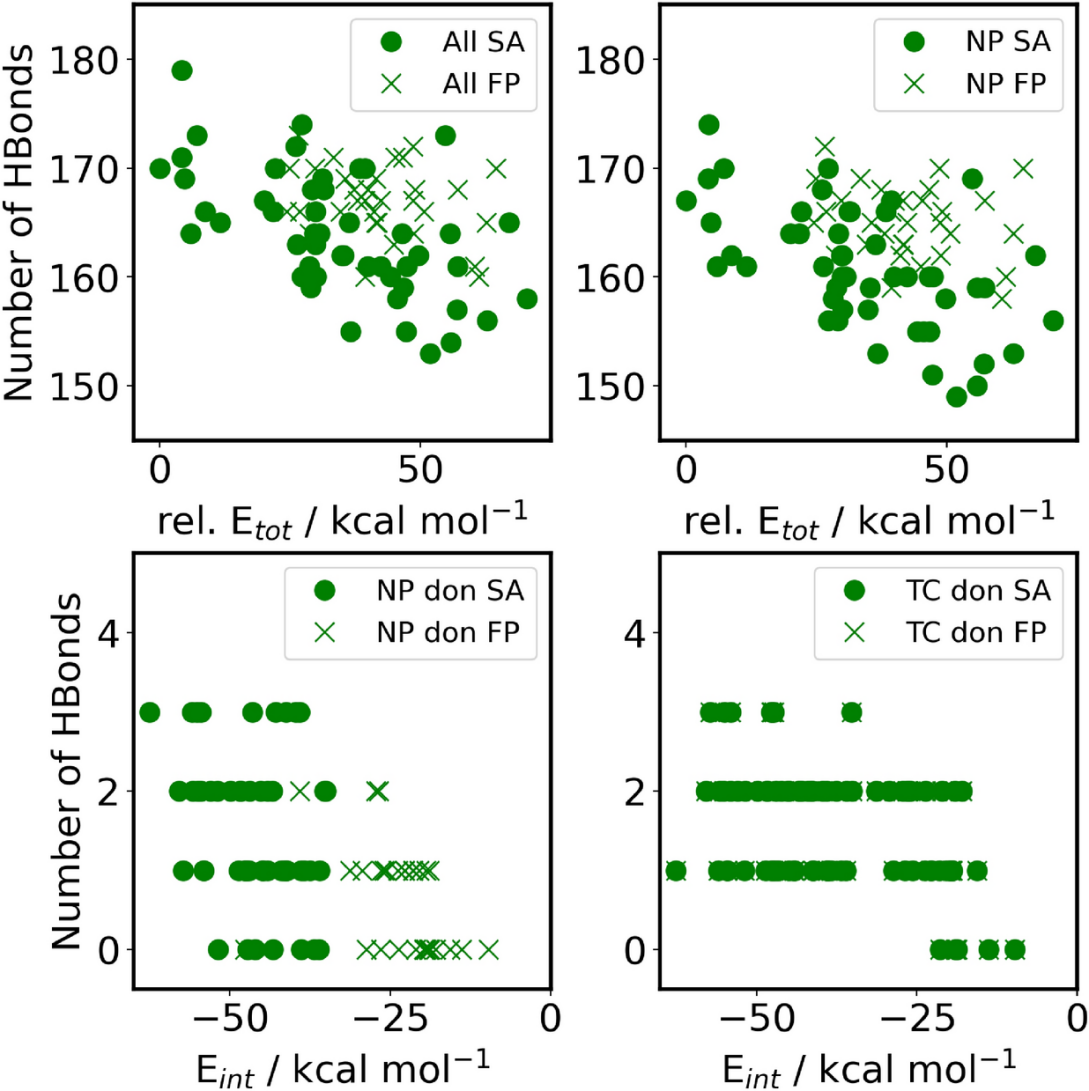
Number of hydrogen bonds in TC@N66 against energies. Each symbol represents an aggregate conformer. Upper left: All hydrogen bonds in the system versus relative total energies. Upper right: All hydrogen donated and accepted by the NP versus relative total energies. Lower left: All hydrogen bonds accepted by the TC molecules versus interaction energies. Lower right: All hydrogen bonds donated by the TC molecule versus interaction energies. (Hydrogen bonds against the index see Supplementary Fig. S8.

Similarly, the different conformations of a N66 polymer chain should exhibit significant barriers since the rearrangement between them may necessitate breaking hydrogen bonds between the amide groups. Thisnot only makes the SA approach more likely to result in a higher energy structure but also makes the differences in the relative energies between the various conformations larger, as a single hydrogen bond can be more than 10 kcal mol$^{-1}$ in strength. Hydrogen bonds can be observed and counted within the structures by defining a distance criterion (3.5 Å) and an angle N–H$\cdots$O cutoff (30$^{\circ }$), exhibiting total numbers between 150 and 180. These values show a common trend with the total energy of the structure. However, there are apparently other effects at play (Fig. 4), which prompted us to refrain from a linear regression analysis. Furthermore, although the repeating sequences of the hydrocarbon and amide moieties create a well-defined surface, they also make different sites that can interact with the tetracycline with varying strengths. Thus, the number of hydrogen bonds between the drug and the nanoplastic varies greatly, strongly affecting the interaction energy between the two compounds (see right side of Fig. 3 and Table 3).

**Table 3:** Interaction energy: collection of mean, standard deviation ($\sigma$), skewness (skew), kurtosis (kurt), x-median (xMed), lowest x-value (xlow) and highest x-value (xhi) for histogram and bootstrapped histogram. SA: simulated annealing approach; SA$^{B}$: SA bootstrapped; FP: fixed particle approach; FP$^{B}$: fixed particle bootstrapped.

	PE				PP			
	SA	SA$^B$	FP	FP$^B$	SA	SA$^B$	FP	FP$^B$
Mean	−22.0	−22.0	−11.8	−11.8	−21.1	−21.1	−12.3	−12.3
$\sigma$	2.4	0.3	2.6	0.4	2.7	0.4	2.8	0.4
95 % conf.	0.7	–	0.8	–	0.8	–	0.9	–
skew	1.07	0.16	−0.29	−0.02	−0.06	−0.02	−0.75	−0.12
kurt	2.12	0.00	−0.24	0.03	0.37	0.02	0.20	−0.02
xMed	−22.2	−22.0	−11.5	−11.8	−21.0	−21.1	−12.0	−12.3
xlow	−25.9	−23.1	−18.5	−13.2	−27.9	−22.5	−19.7	−14.1
xhi	−13.3	−20.5	−6.2	−10.2	−15.1	−19.5	−8.2	−10.8

	PS				N66			
	SA	SA$^B$	FP	FP$^B$	SA	SA$^B$	FP	FP$^B$
Mean	−26.4	−26.4	−15.2	−15.2	−46	−46	−23	−23
$\sigma$	2.9	0.4	2.9	0.4	7	1	7	1
95 % conf.	0.8	–	0.8	–	2	–	3	–
skew	0.36	0.08	−0.03	0.00	−0.23	−0.03	−1.41	−0.24
kurt	−0.70	0.03	−0.03	−0.01	−0.79	−0.03	3.27	0.08
xMed	−27.1	−26.4	−14.9	−15.2	−46	−46	−21	−23
xlow	−31.5	−28.1	−22.6	−16.7	−63	−50	−48	−29
xhi	−19.3	−24.8	−8.3	−13.8	−35	−43	−10	−19

A lower scatter in the relative total energies was found since the same structure was taken for each plastic for FP (Fig. 3). However, as the plastic particle cannot adjust to the drug, and hence the drug cannot enter the particle, using a pre-folded particle to study drug-plastic interplay can lead to an underestimation of the stability of the corresponding complexes and the strength of the interaction. In contrast, when refolding the plastic structure in the presence of the TC, the polymer chains can adjust to the TC molecule, and choose the best possible conformation to maximize the sum of NP-NP and NP-TC interplay. This difference can be clearly demonstrated via the example of the interaction of the N66 particle with TC. The FP approach has a significantly higher propensity of creating structures with fewer hydrogen bonds between drug and plastic, yielding substantially less stable structures than the SA method (Fig. 4). According to the data in Table 2, it is evident that refolding the plastic to let it adjust to the drug produces significantly more stable structures, with differences between the most stable structures from the SA and FP approaches of up to 66.7 kcal mol$^{-1}$. It should be, however, also mentioned here that the sizable difference in total energies between the structures obtained through these two approaches partly stems from the reference structure in the FP method. We selected the folded plastic structure that exhibited the lowest energy according to the molecular mechanical force field calculations at the end of the energy minimization after the simulated annealing of the plastic. However, the semi-empirical quantum chemical energies, after geometry optimization, showed a slightly different trend, shifting the reference energies significantly. However, the interaction energies—used as reference the neat plastic particle with an identical geometry to that within the complex—also indicate a superior performance of the SA approach.

Analyzing the structures and correlating plastic-drug interaction energies and the total relative energies with the way TC is situated on the NP provide essential information on how this antibiotic prefers being adsorbed on the plastic surfaces considered here. Tetracycline has multiple polar functional groups, concentrated predominantly on one side of the molecule, see Fig. 1. This almost amphiphilic structure determines the modes of interaction between TC and the NP. On polyethylene, the less polar side of the molecule is attached to the plastic, while the carbonyl, hydroxyl and amide groups point toward the aqueous solvent. On N66, the alignment of TC is opposite, and the polar-polar interaction between the particle and the antibiotic is apparently stronger than the solvation of these very groups.

**Fig. 5:**
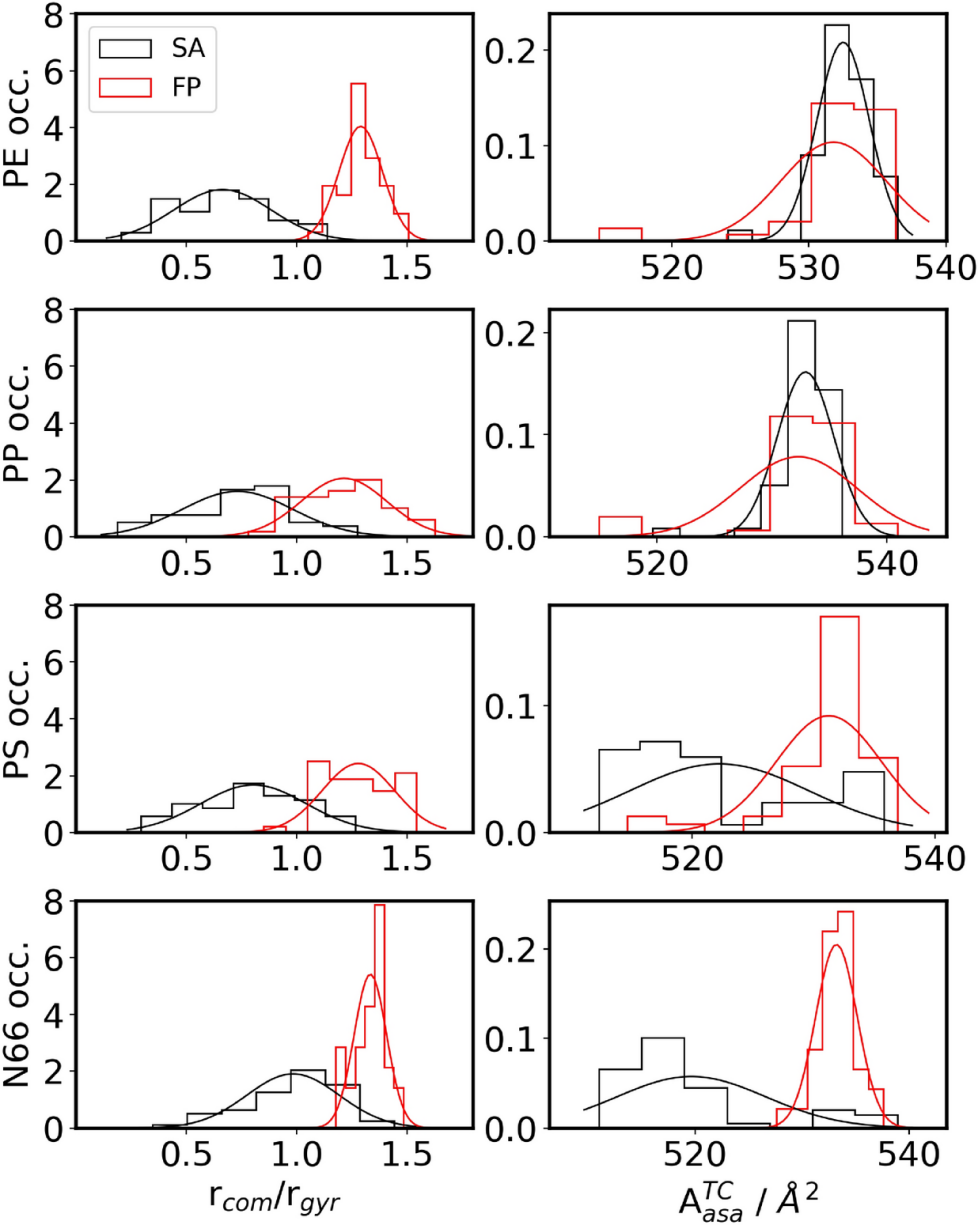
Left: Occurrence of ratio of center of mass distance (TC to NP) divided by the radius of gyration of the NP. Right: Occurrence of accessible surface area A$_{asa}^{TC}$ for TC. The histograms are done with bins of 7 according to Sturge’s rule. The normal distribution plots are only given to guide the eye.

It is interesting to point out that the drug molecule in the SA approach, which produces the more stable TC@NP structures, the drug molecule is often situated “inside” the particle, buried beneath the polymer chains. A similar observation was made by molecular dynamics simulations of a peptide adsorbed on the surface of a PE nanoparticle. In these simulations, the polymer chains rearranged so that they clear a path for the peptide to move toward the center of the NP^52^. The driving force for the SA to produce such conformations should be that if the polymer surrounds the tetracycline, they interact through a larger interface, stabilizing the structure, provided that the drug-plastic interactions can compensate for the disrupted plastic-plastic interplay. This finding is in line with the swelling of plastic surfaces in hydrophobic media, which involves the solvent molecules penetrating the interfacial region of the polymer, loosening the entangled structure of the plastic.

If a molecule, adsorbed onto the surface of a nanoplastic particle, can diffuse in between the polymer chains, this would have significant consequences. In case of the most frequently investigated nanoparticles—built from e.g. metals, metal oxides, silicates—it has been discussed that their composition does not necessarily play a pivotal role in their toxicity, as its environment, for instance a cell may sense and respond to only the molecules that cover the surface, i.e., the corona^72,73^. Plastic nanoparticles possess, however, a property that makes them significantly different from materials mentioned above, namely that they are composed of polymer chains that may be associated only through weak secondary intermolecular forces. Thus, instead of having a distinct border between the particle and its corona, these two materials may mix, forming a heterogeneous droplet composed of a mixture of multiple compounds. Furthermore, the capacity of a nanoparticle to carry environmental molecules—e.g. drugs, endocrine disruptors, heavy metals, pesticides—should be higher, if the whole volume of the species is accessible for these compounds rather than just its surface.

To quantify this effect, we defined the ratio of the drug-plastic centers of mass distance ($r_{com}$) and the radius of gyration ($r_{gyr}$), see Fig. 5 left. Assuming a perfect, homogeneous sphere, this ratio converges to a value of $r_{com}/r_{gyr} = 1/\sqrt{3/5} \approx 1.29$ for a particle sitting directly on the surface. However, due to the NPs rough surface structure and empty spaces within the particle, $r_{gyr}$ is generally larger for all investigated structures. Additionally, the $r_{com}$ does not contain information regarding the orientation of the TC with respect to the NP. Therefore, the ratio has certain limitations in its accuracy to predict the TC position, but from visual observation of the analyzed structures, we found that the values of $r_{com}/r_{gyr}$, for structures with the TC positioned on a NPs surface, mostly lie in a range of 1–1.4. As a crude approximation and for simplicity we decided to characterize structures with ratio below 1 as the TC being inside the NP. For all plastic particles we observe that the scatter for the SA method is wider than that for the FP approach, which is particularly the case for PE and N66. Supplementary Table S3 shows the corresponding statistics. Additionally, the FP approach, apparent for obvious reasons, shows higher values of the ratio, i.e., the conformers do not show the TC molecule inside of the NP as opposed to the SA conformers. Interestingly, and in contradiction to the very hydrophilic nature, the TC can enter the PE and the PP in more cases than the N66 where the TC molecule is found closer to the surface than deep inside of the NP, i.e., the mean of the ratio is 1 for N66 and 0.7 for PE as well as for the PP.

**Fig. 6:**
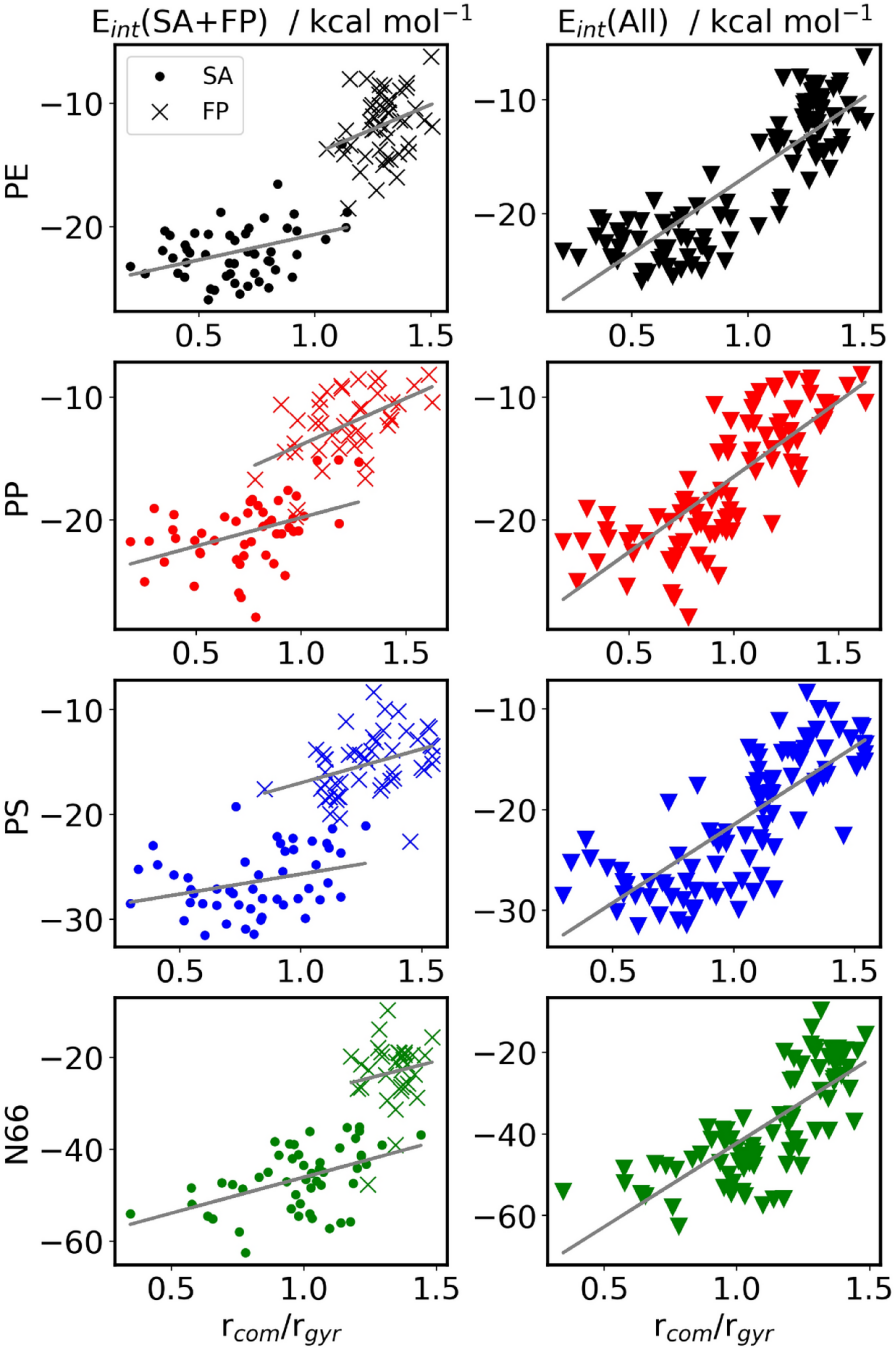
The ratio of center of mass TC versus NP and the radius of gyration plotted against the interaction energy.

When plotting the interaction energies ($\Delta E_{int}$) against $r_{com}/r_{gyr}$ for all the structures obtained, it becomes apparent that there is a notable, albeit somewhat weak correlation between these two quantities, with a stronger interplay when the drug molecule is inside the plastic particle (Fig. 6). As approaching from the larger $r_{com}/r_{gyr}$ values—i.e., from the structures with the TC excluded from the plastic particle—the slope of this correlation is quite large. Thus, if the TC is closer to the surface, the energy decreases significantly, as the drug/plastic interface grows. However, when the $r_{com}/r_{gyr}=1$ value is reached, the slope becomes smaller, since from this point on the plastic has already covered most of the antibiotic, and therefore burying this molecule deeper into the particle does not produce significant further stabilization. Thus, in the structures with the TC inside the plastic, the energy of the system will probably exclusively depend on the efficiency of the polymer chains’ packing. In agreement, the total energies of these structures exhibit a different trend, and in contrast to the $\Delta E_{int}$, the structures with the minimal $\Delta E_{tot}$ do not necessarily correspond to structures with low $r_{com}/r_{gyr}$ ratios (see Supplementary Fig. S12).

After observing this common trend between interaction energy and $r_\textrm{com} / r_\textrm{gyr}$ ratio, and the finding that the more the TC is inside the NP the stronger it interacts with the plastic, the accessible surface area (A$_{asa}^{TC}$) of the TC puts these results into a different perspective. Figure 5 (right) plots this measure for all plastic particles. For the FP approach, all particles behave similarly, with a mean value of 533 Å$^2$ (see Supplementary Table S4 and Supplementary Fig. S13). This value also coincides with that obtained from the SA approach in case of PE and PP. These are the NPs, in which the TC is buried most. For PS and N66, however, lower average A$_{asa}^{TC}$ values were found with 522 and 520 Å$^2$ , indicating that the antibiotic adopts a more compact conformation, associated with a higher reorganization energy $\Delta E_{reorg}^{TC}$ (see Table 4 for the values of the most stable TC-NP aggregates), to maximize its interaction with the given NP.

While TC clearly penetrates less into N66 and remains closer to the surface, the average accessible surface area of TC is the smallest in these complexes (cf. Fig. 5 left and right), highlighting that the driving force behind the more compact conformation is to maximize the favorable interactions with the NPs and is not caused by the embedded position within the NPs. This implies that in case of PE and PP, the TC is in a relatively large void, arising from packing effects within the NP.

The interaction energies ($\Delta E_{int}$, see Fig. 6 and Table 4) were found similar for the three polyolefins PE, PP, and PS, as they are all similar in interacting with tetracycline solely through weak van der Waals forces. The strongest interaction can be observed for N66, which—as discussed above—offers a multitude of hydrogen bond donor and acceptor sites to the antibiotic (Fig. 4). However, rearranging the polymer chains in this plastic to make the cavity for the drug requires more energy due to the strong interactions between the amide moieties of N66, making the structure with TC attached to the surface, rather than embedded, energetically more favorable. In the case of PP and PS, the substituents that are attached to the backbone of the polymer chain hinder these chains from being packed very close to each other, leaving significant voids in the structure of the plastic itself. In the presence of these cavities, the reorganization of the plastic in the adsorption process is less demanding energetically compared to PE.

**Table 4:** Interaction energies ($\Delta E_{int}$), reorganization energies for the plastic particle ($\Delta E_{reorg}^{NP}$) and tetracycline ($\Delta E_{reorg}^{TC}$), sorption energies ($\Delta E_{ads}$) and accessible surface area of tetracycline ($A_{asa}^{TC}$) for the most stable obtained tetracycline-nanoplastic aggregates. All energies are given in kcal mol$^{-1}$.

Plastic	$\Delta E_{int}$	$\Delta E_{reorg}^{NP}$	$\Delta E_{reorg}^{TC}$	$\Delta E_{ads}$	$A_{asa}^{TC}$
PE	−23.2	8.7	10.3	−4.3	530
PP	−22.0	1.0	10.3	−10.6	536
PS	−25.2	1.6	12.0	−11.6	518
N66	−36.0	1.9	15.0	−19.1	518

The combination of the effects above results in the adsorption energies ($\Delta E_{ads}$) presented in Table 4. Interestingly, although the $\Delta E_{int}$ data were similar for all polyolefins, the $\Delta E_{ads}$ values were more negative for PP and PS, due to the lower reorganization energy of the plastic upon adsorption. The high $\Delta E_{int}$ found in the case of N66 yields the most negative adsorption of the four hereby investigated plastic materials.

### Tetracycline on and in nanoplastics solvated in water

**Fig. 7:**
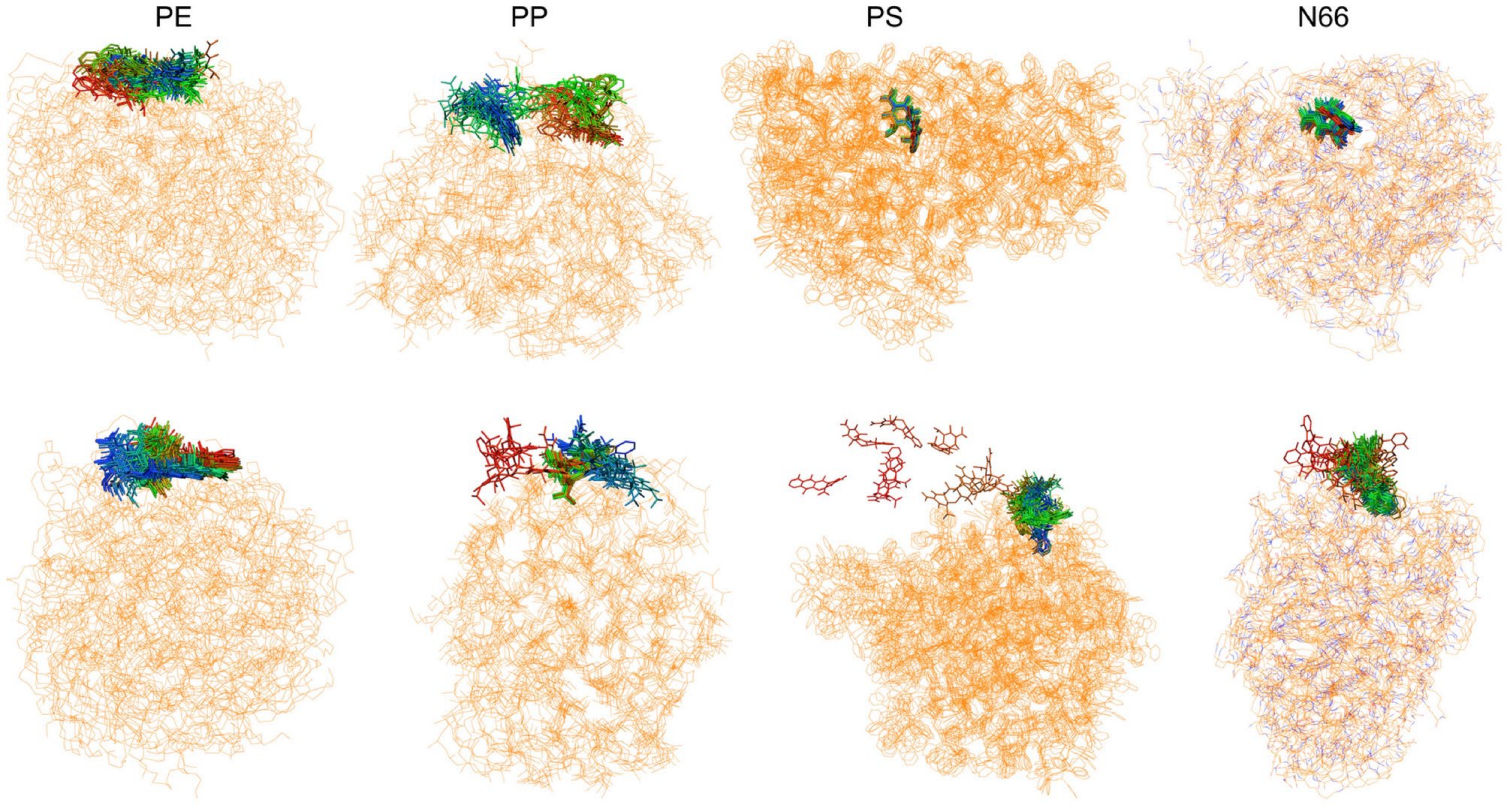
Snapshots of the MD simulations of the tetracycline-plastic systems. 100 snapshots are overlapped with the tetracycline shown in color-changing-with-time. Water molecules are omitted for clarity. Start: Red, Middle: Green; and End: Blue; Above: trajectory starting from a small $r_\textrm{com} / r_\textrm{gyr}$ ratio; Below: trajectory starting from a large $r_\textrm{com} / r_\textrm{gyr}$ ratio.

Based on the SA and FP calculations, we found that the present drug-polymer systems form low-energy aggregates with the tetracycline, with thermodynamically favorable adsorption. Moreover, the drug often prefers being inside the plastic particle, fully covered by the polymer chains. For the formation of these conformations, the polymer chains must be mobile enough for the antibiotic to penetrate the particle. Therefore, we investigated two TC@NP structures of each type of plastic in water using molecular dynamics simulations to gain preliminary knowledge about their stability concerning temperature and solvation. The investigated structures were selected from the SA approach, with the two structures of each plastic being those with the highest and lowest $r_\textrm{com} / r_\textrm{gyr}$ ratio, of all structures lying within 20 kcal mol$^{-1}$ of the energetically best TC@NP structure. We paid special attention to the solvation of these aggregates, and the mobility of the molecules they are comprise.

The mobility of the chains can be expressed through the root mean square deviations (RMSDs, Supplementary Fig. S16), which describe how much the atoms of the TC@NP system rearrange over time. It is visible that the plastics exhibit significant differences in their mobilities. The least mobile structures are PS chains, which have the largest functionalities attached to the polyolefin backbone that can provide significant friction or steric hindrance in their rearrangement. Similarly, the movement of the N66 macromolecules is hindered by the strong hydrogen bonds between the amide groups, although the hydrocarbon units in between may have some mobility. The rearrangement of PE is somewhat more significant, but that of PP is remarkable, showing almost two times larger deviations with respect to the starting structures.

These results show that while the simulation time is limited compared to the time scale of macromolecular motion, at least PP is capable of rearranging enough to accommodate drug molecules within, e.g. tetracycline. In case of this particular drug, however, radial diffusion can occur in both directions. In case of the low $r_\textrm{com} / r_\textrm{gyr}$ ratio simulations with PE and PP, the moieties that covered TC, and separated it from the aqueous solvent, rearranged in a way that TC was uncovered already during the equilibration phase. This change allowed the polar, hydrophillic substituents of TC to interact with the water molecules, resulting in the stabilization of the system.

**Table 5:** The average hydrogen bond number with AD/A/D indicates acceptor and donor hydrogen bonds from the TC molecule to water. First value: trajectory starting from the small $r_\textrm{com} / r_\textrm{gyr}$ ratio; Second value: trajectory starting from the large $r_\textrm{com} / r_\textrm{gyr}$ ratio.

Plastic	AD		A		D	
PE	5.334	5.452	3.828	3.976	1.506	1.476
PP	5.428	6.034	3.876	4.394	1.552	1.640
PS	0.268	4.872	0.030	3.444	0.238	1.428
N66	0.896	4.814	0.884	3.960	0.012	0.854

In agreement, plotting the radial position of the TC in or on the NP ($r_{COM}$) as a function of time shows that the drug diffuses significantly on top of the PP and N66 particles (see Supplementary Fig. S17). It is also notable that in case of PS, the drug detached from the surface during the equilibration. However, it found its way back to the plastic over the course of the subsequent production run, apparently finding a stronger binding site. The fact that the drug was spontaneously adsorbed on the PS represents the affinity of these compounds toward each other, indicating that the complexes obtained here are not metastable states.

Interestingly, except for PS, the $r_\textrm{gyr}$ values also change significantly for the particles, especially with small $r_\textrm{com} / r_\textrm{gyr}$ ratio (see Supplementary Fig. S17). Over the course of the simulation, these systems seem to shrink, obtaining a more compact packing of the polymer chains. This process appears to be slower than the range of the present simulations, and would presumably continue after the 50 ns of physical time that was simulated here.

Considering the $r_\textrm{com} / r_\textrm{gyr}$ ratios, it is visible that the shrinking of the particles and the radial movement of the TC results in an overall rearrangement of the TC@NP complex. For most plastics, this mobility is more intensive in the first quarter of the simulation. The movement of the TC can be shown by overlaying its position through the 50 ns run (Fig. 7). It is clearly visible that the TC molecule rearranges and drifts notably. In case of PP, this movement is especially striking, in accordance with the aforementioned dynamics of the TC@NP complex.

The dynamics of the system can also be observed by the development of the number of hydrogen bonding interactions between the TC and the neighboring water molecules over time (see Table 5 and Supplementary Fig. S18). In case of the simulations with the TC at the surface—i.e., with large $r_\textrm{com} / r_\textrm{gyr}$ ratio—the number of hydrogen bonds is high, many of the hydrogen bond donor and acceptor sites are occupied with a water molecule. In case of the PS particle, the re-adsorption of TC can be followed through the number of hydrogen bonds, gradually as the plastic prevents access of the water molecules to the drug. This suggests that via the alignment of the TC at the surface of the plastic, the polymer chains rearrange so that the drug is covered more and more as the simulation progresses further.

For the simulations, starting from the antibiotic within the plastic particle—i.e., having a low $r_\textrm{com} / r_\textrm{gyr}$ ratio—significant differences can be observed. N66 and PS keep the TC inside the particle, and therefore, water molecules cannot approach the hydrogen bonding sites of the drug (see Supplementary Fig. S18). Furthermore, the hydrogen bonding sites of N66 can form interactions with TC, as discussed above; hence, they effectively compete with water for the antibiotics. In the case of PE and PP, as the polymer chains make way for the water molecules already during the equilibration phase, the number of hydrogen bonding interactions between the solvent and the drug is similarly high to that in the simulations starting from structures with the TC on the surface of the plastic (see Supplementary Fig. S18).

### Experimental proof for the relevance of tetracycline-plastic interactions in biological media

**Fig. 8:**
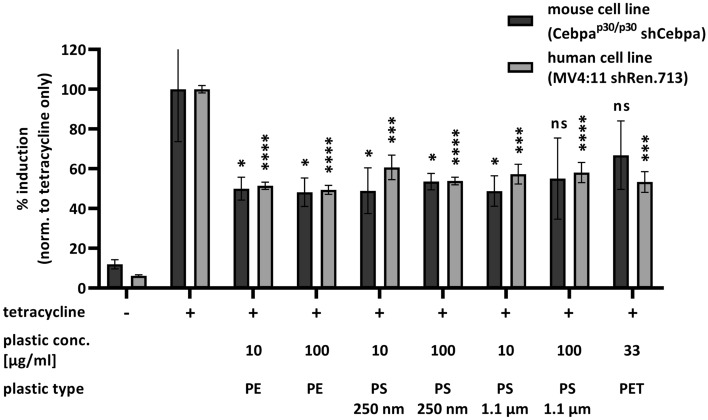
Effects of polystyrene (PS, 250 nm or 1.1 $\upmu$m mean particle size), polyethylene (PE, size range 0.2–9.9 $\upmu$m) or polyethylene terephthalate (PET, 1.9 $\upmu$m mean particle size) microplastic particles at given concentrations on tetracycline-induced expression of a dsRed or IRFP670 fluorescent reporter in mouse or human cells, respectively. The percentage of dsRed or IRFP670 induction was normalized to the effects of tetracycline only [16 $\upmu$g/ml] (n = 3). Statistical significance *p < 0.05, ***p < 0.001, ****p < 0.0001 was assessed by a two-tailed t-test. ns not significant.

Earlier experiments showed that dissolved organic matter decreases the ability of MNPs to adsorb TC^45,49^. However, in a more complex matrix that is present in biological systems, the intricate network of intermolecular interactions can cancel such effects, and despite the presence of organic substances, a significant amount of TC is adsorbed by the MNP. Thus, to test changes induced by microplastics in the cellular effect of tetracycline, we used two cell line models in which tetracycline-induced gene expression can be measured via the induction of a fluorescent reporter. We found that incubation of cells with all three types of microplastics tested (PS, PE and PET) caused a significant reduction of the tetracycline-induced expression (40–50%) of the reporter protein in both human and mouse cell lines after 45 h (Fig. 8).

## Summary and conclusion

In this work, the interaction of nanoplastics with biologically active compounds was investigated, on the example of the antibiotic drug tetracycline, through theoretical chemical methods and in vitro experiments. We chose two alternative strategies to generate thermodynamically relevant conformations, both relying on simulated annealing molecular dynamics. In the first approach (SA), the plastic was folded through simulated annealing in the presence of the tetracycline. Since the folding, due to the limited simulation time of the annealing, may result in high energy structures, we performed this process 50 times for each plastic-tetracycline system from different starting geometries. In the other strategy (FP), we first folded the neat plastic, and then placed the tetracycline in several orientations onto the surface of the best structure found, followed by a short molecular dynamics run. The 104 obtained structures from the two methods underwent a semiempirical quantum chemical geometry optimization with an implicit solvent model, which gave the accessible most accurate relative energies for the different geometries.

The difference between the two approaches becomes apparent when analyzing the structure and energy of the aggregates obtained. While folding the plastic in the presence of the tetracycline can result in high energy structures, it allows the reorientation and adjustment of the polymer chains to the drug. It is, therefore, not bound by the starting geometry of the plastic particle. For all plastics, the SA method produced the most stable plastic-tetracycline complexes. Even more interestingly, in many low energy structures the drug was situated inside the plastic particle, suggesting that such compounds can be bound not only onto the surface of nanoplastics but can diffuse in between the chains. Such a process should be similar to the swelling of plastic surfaces and polymer network in solvents. The experimentally observed morphological changes of MNPs upon adsorption of TC, reported recently, appear to support this finding^48^. These results clearly suggest the superiority of the SA approach over the FP. Therefore, in computational studies, we recommend using this method to create the most stable starting structures.

The resulting adsorption energies show a clear trend, indicating an affinity to the drug decreasing in the order of N66> PS> PP > PE, in very good qualitative agreement with earlier experimental observations^45^. The values themselves are between $\Delta E_{ads} = -4.3$ and $-19.1$ kJ mol$^{-1}$. These values suggest that the adsorption can be in the range that is exothermic enough to make a physical difference in a suspension having both micro- and nanoplastics and this particular drug, but not too exothermic, hence the drug can be delivered from an external source and released in the human body. Verifying the computational results above, experiments with cell line models were conducted, based on tetracycline-induced gene expression. The experiments showed that the effect of tetracycline on the cells decreased significantly in the presence of the plastics.

We performed equilibrium molecular dynamics on selected low-energy structures to observe if the complexes produced in the methods above are stable in terms of temperature and water solvation. These simulations confirmed the affinity of the plastics to the antibiotic, and showed that the chains rearrange already within 50 ns. Furthermore, the tetracycline molecule also appears to drift on the nanoplastic particle’s surface. These findings suggest that the mobility of both the plastic and the drug is high enough to allow the radial diffusion of the adsorbed molecule into the inner domain of the particle.

The results above from the simulated annealing, quantum chemistry, and experimental data clearly indicate that the interaction of micro- and nanoplastics with drugs can significantly impact on the environment and human health through multiple mechanisms. First, as shown by the experiments, the absorption of the drug into the human body can be altered if they are ingested together with plastics. Second, the adsorption energies indicate that the adsorption to the plastic is reversible, and the drug may be carried and delivered at a different location later, perhaps even in a different organism. Third, due to the adsorption’s thermodynamically favourable nature, the local concentration of antibiotics can be increased at the surface of the particles, which may induce the development of antibiotic-resistant bacterial strains. This is especially the case for those plastics that exhibit more affinity to the given drug, collecting it more efficiently from the given matrix, even at lower concentrations. All these effects are enhanced by the hereby observed effect that the adsorbed molecule—through swelling its polymeric network—can penetrate the plastic particle, making thereby much of its volume available to accommodate the adsorbed molecule than just the immediate surface area. In summary, the results above show that MNPs can pose a variety of direct health risks, as they can carry unwanted drugs into the body, may interfere with medical therapy, and can contribute to the development of antibiotic resistant strains of bacteria.

## Supplementary Information

Supplementary Information 1.

Supplementary Information 2.

## Data Availability

All generated geometry-optimized NP and TC@NP structures are publicly available. The molecular dynamics trajectories analyzed for this study are available on request from O.H. or B.K. due to their size. All data sets resulting from the analyses of the geometrically optimized structures and the MD trajectories are also publicly available and stored on zenodo (10.5281/zenodo.12167648).
