# Supplementary material for: The adsorption of drugs on nanoplastics has severe biological impact
Source: Sci Rep. 2024 Oct 28;14:25853. doi: 10.1038/s41598-024-75785-4 (PMC11519658; doi:10.1038/s41598-024-75785-4)
Supplement: Supplementary file 1 — Supplementary Information 1. [file 41598_2024_75785_MOESM1_ESM.pdf]

The adsorption of drugs on nanoplastics has severe biological impact

Leonard Dick[1,2], Patrick R. Batista[1,3], Paul Zaby[1], Gabriele Manhart[4,5], Verena Kopatz[6,7,8,9], Lukas Kogler[7,10,11], Verena Pichler[7,11], Florian Grebien[4,5,12], Vince Bakos[13,14], Benedek G. Plósz[13], Nikola Zlatkov Kolev[15], Lukas Kenner[6,7,9,16,17*], Barbara Kirchner[1*], and Oldamur Hollóczki[18*]

[1]Mulliken Center for Theoretical Chemistry, University of Bonn, Beringstr. 4+6, D-53115 Bonn, Germany
[2]Department of Colloid Chemistry, Max Planck Institute of Colloids and Interfaces, Am Mühlenberg 1, D-14476 Potsdam, Germany
[3]Institute of Chemistry, University of Campinas, Monteiro Lobato, 270, Cidade Universitária, 13083-862, Campinas, São Paulo, Brazil
[4]Medical Biochemistry, Dept for Biological Sciences and Pathobiology, University of Veterinary Medicine Vienna, 1210 Vienna, Austria
[5]CeMM Research Center for Molecular Medicine of the Austrian Academy of Sciences, 1090 Vienna, Austria
[6]Medical University of Vienna, Clinical Institute of Pathology, Department for Experimental and Laboratory Animal Pathology, 1090 Vienna, Austria
[7]Center for Biomarker Research in Medicine (CBmed GmBH), microOne, 8010 Graz, Austria
[8]Department for Radiation Oncology, Medical University of Vienna, 1210 Vienna, Austria
[9]Comprehensive Cancer Center Vienna, Medical University of Vienna, 1090 Vienna, Austria
[10]Department of Biomedical Imaging and Image-guided Therapy, Medical University of Vienna, 1090 Vienna, Austria
[11]Division of Pharmaceutical Chemistry, University of Vienna, 1090 Vienna, Austria
[12]St. Anna Children‘s Cancer Research Institute (CCRI), 1090 Vienna, Austria
[13]Department of Chemical Engineering, University of Bath, Claverton Down, Bath BA2 7AY, United Kingdom
[14]Department of Applied Biotechnology and Food Science, Budapest University of Technology and Economics, 1111 Budapest, Műegyetem rkp. 3, Hungary
[15]Department of Molecular Biology, Umeå University, Umeå, Sweden
[16]Christian Doppler Laboratory for Applied Metabolomics, Medical University of Vienna, 1090 Vienna, Austria
[17]Unit of Laboratory Animal Pathology, University of Veterinary Medicine Vienna, 1210 Vienna, Austria
[18]Department of Physical Chemistry, Faculty of Science and Technology, University of Debrecen, Egyetem tér 1, H-4032 Debrecen, Hungary
*holloczki.oldamur@science.unideb.hu, lukas.kenner@meduniwien.ac.at, kirchner@thch.uni-bonn.de

ABSTRACT

Micro- and nanoplastics can interact with various biologically active compounds forming aggregates of which the effects have yet to be understood. To this end, it is vital to characterize these aggregates of key compounds and micro- and nanoplastics. In this study, we examined the adsorption of the antibiotic tetracycline on four different nanoplastics, made of polyethylene (PE), polypropylene (PP), polystyrene (PS), and nylon 6,6 (N66) through chemical computation. Two separate approaches were employed to generate relevant conformations of the tetracycline-plastic complexes. In the first approach, we folded the plastic particle from individual polymer chains in the presence of the drug through multiple separate simulated annealing setups. In the second, more biased, approach, the neat plastic was pre-folded through simulated annealing, and the drug was placed at its surface in multiple orientations. The former approach was clearly superior to the other, obtaining lower energy conformations even with the antibiotic buried inside the plastic particle. Quantum chemical calculations on the structures revealed that the adsorption energies show a trend of of decreasing affinity to the drug in the order of N66 > PS > PP > PE. In vitro experiments on tetracycline-sensitive cell lines demonstrated that, in qualitative agreement with the calculations, the biological activity of tetracycline drops significantly in the presence of PS particles. Preliminary molecular dynamics simulations on two selected aggregates with each plastic served as first stability test of the aggregates under influence of temperature and in water. We found that all the selected cases persisted in water indicating that the aggregates may be stable also in more realistic environments. In summary, our data show that the interaction of micro- and nanoplastics with drugs can alter drug absorption, facilitate drug transport to new locations, and increase local antibiotic concentrations, potentially attenuating antibiotic effect and at the same time promoting antibiotic resistance.

List of Figures

S1 Scheme showing the creation of a series of geometries for the tetracycline-nanoplastic complexes. S3
S2 Computational protocol flowchart. S4
S3 Definitions of the characteristic energy terms. S5
S4 Total energy referenced to the global minimum value. S6
S5 Total energy referenced to the global minimum value with all structures from SA and FP sorted together. S7
S6 Total energy referenced to the respective minimum value. S7
S7 Interaction energy for the different TC@NP complexes S8
S8 Analysis of hydrogen bonds in the TC@N66 systems. S9
S9 Radius of gyration and center of mass distances versus index for all four TC@NP systems S9
S10 Center of mass distance plotted against the index. S10
S11 Ratio of the center of mass distance r_{com} (TC–NP) divided by the radius of gyration r_{gyr} of the NP. S11
S12 Ratio of the center of mass distance r_{com} (TC–NP) divided by the radius of gyration r_{gyr} of the NP with SA and FP sorted together. S11
S13 Accessible surface area of isolated TC molecules (A_{asa}^{TC}) S12
S14 Accessible surface area plotted for all investigated TC@NP structures for the isolated TC (A_{asa}^{TC}) and the isolated NP (A_{asa}^{NP}). Interaction energies (E_{int}) and r_{com}/r_{gyr} ratio plotted against the accessible surface area of isolated TC molecules A_{asa}^{TC} for all TC@NP structures. S13
S15 Accessible surface area (A_{asa}^{TC}) and respective volume (V^{TC}) of isolated TC molecules plotted for all investigated TC@NP structures. S13
S16 Root mean square deviation for the NP and root mean square deviation of TC@NP complexes. S15
S17 Center of mass distance (TC–NP), radius of gyration of NP and ratio of the two plotted against the simulation time. S16
S18 Number of hydrogen bonds plotted against the simulation time. S17
S19 Microscopic analysis of in-lab produced PET particles using scanning electron microscopy and light microscopy. S18
S20 FTIR spectra of micro- and nanoplastic particles. S19

List of Tables

S1 Composition of the nanoplastic particles, with the formulas of each chain, and the number of chains per particle. . S3
S2 Cell vectors of all NP and TC@NP systems modeled with 32000 explicit water molecules. S3
S3 Collection of mean, standard deviation (σ), 95 % confidence interval, skewness (skew), kurtosis (kurt), x-median (xMed), lowest x-value (xlow) and highest x-value (xhi) for distribution and bootstrapped distribution of the r_{com}/r_{gyr} ratio for all investigated TC@NP structures. S10
S4 Collection of mean, standard deviation (σ), 95 % confidence interval, skewnesws (skew), kurtosis (kurt), x-median (xMed), lowest x-value (xlow) and highest xvalue (xhi) for distribution and bootstrapped distribution of the accessible surface area of the isolated TC molecules (A_{asa}^{TC}) for all investigated TC@NP structures. S12
S5 Average volume (V_{avg}) and average isoperimetric quotient (Q_{avg}) together with the respective standard deviations (σ_V; σ_Q) for all NP. S16

Computational Details

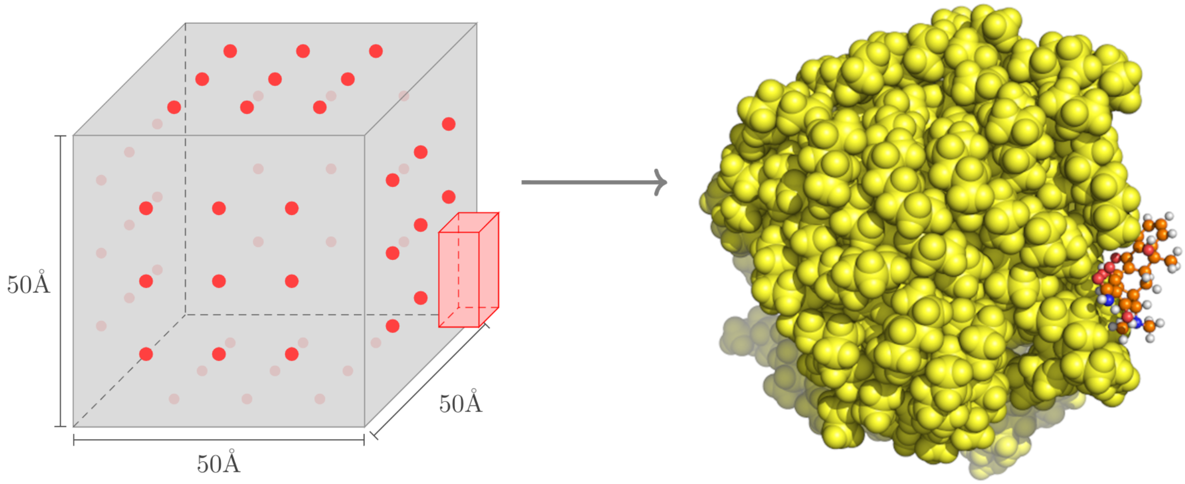

Figure S1. Scheme showing the creation of a series of geometries for the tetracycline-nanoplastic complexes using polyethylene as an example.

Table S1. Composition of the nanoplastic particles studied in this article, with the formulas of each chain, and the number of chains per particle.

Plastic	abbrev.	composition/chain	#chains
Polyethylene	PE	$C_{72}H_{146}$	16
Polypropylene	PP	$C_{144}H_{290}$	8
Polystyrene	PS	$C_{288}H_{290}$	10
Nylon 6,6	N66	$C_{156}H_{288}N_{26}O_{27}$	8

Table S2. Cell vectors of all NP and TC@NP systems modeled with 32000 explicit water molecules.

	cell vector / Å	
Plastic	NP	TC@NP
PE	99.96	99.98
PP	99.94	99.84
PS	101.15	101.15
N66	100.07	100.08

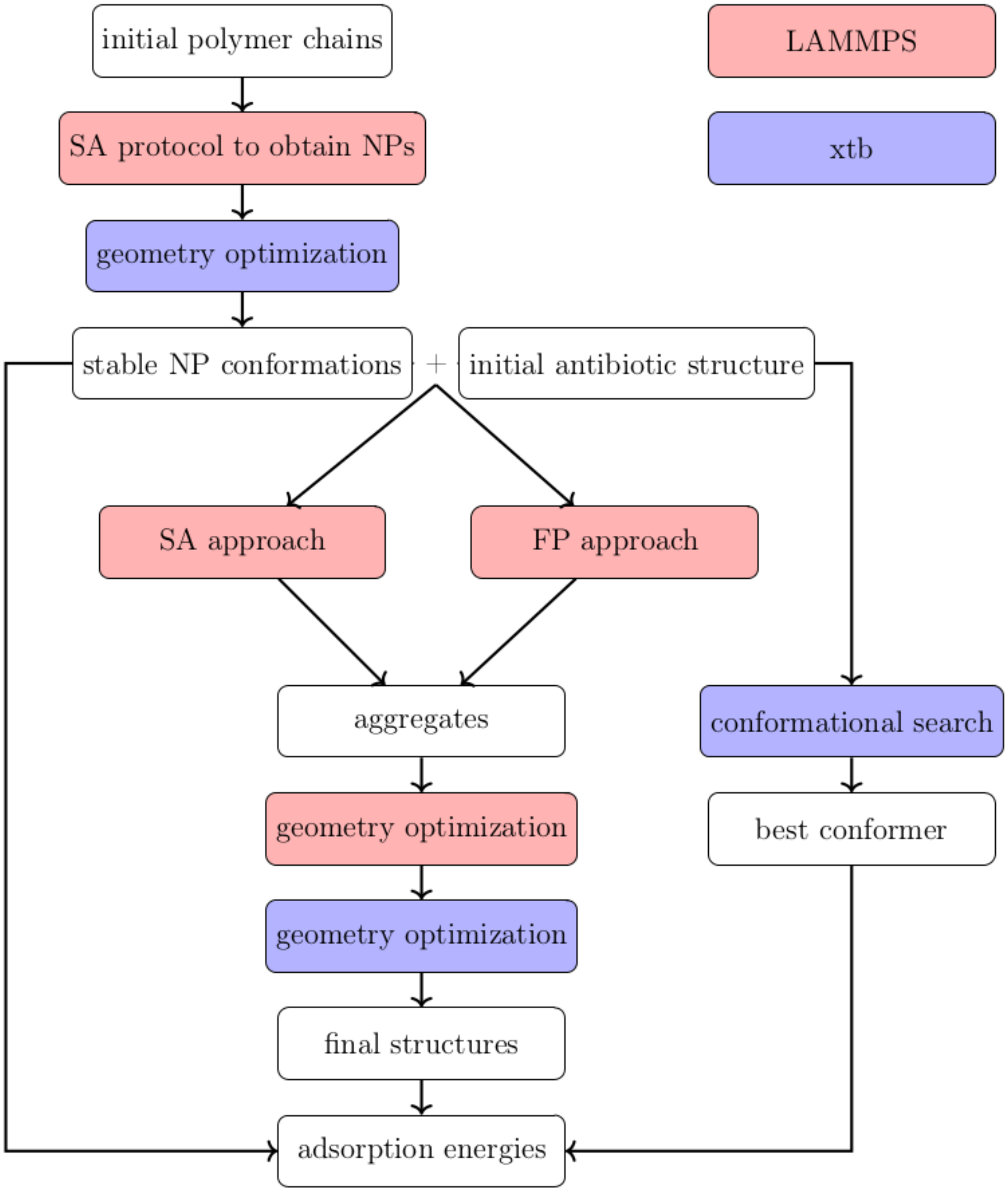

Figure S2. Computational protocol flowchart used to investigate the interaction of nanoplastics with the antibiotic tetracycline.

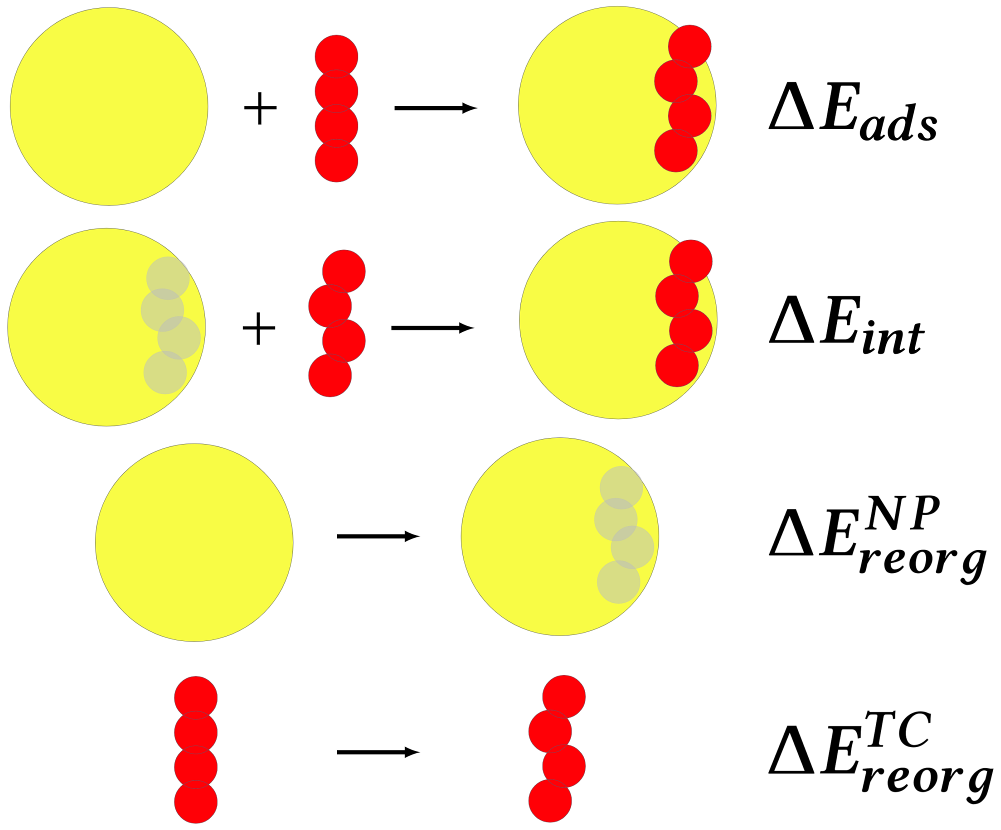

Figure S3. Definitions of the characteristic energy terms to obtain adsorption of tetracycline on the plastics.

Additional results and raw data

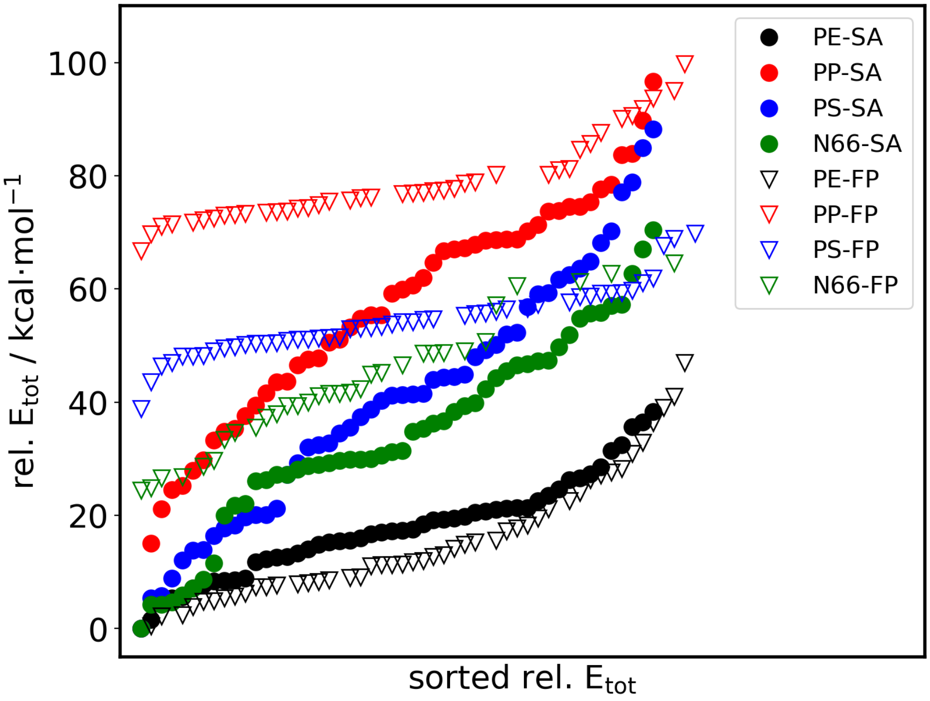

Figure S4. Total energy referenced to the global minimum value in kcal·mol^{-1}. Circles show the simulated annealing approach while open triangles show the FP approach. Note that the reference energy is the lowest energy of all structures from SA and FP together and the data for a given approach is sorted by increasing total energy.

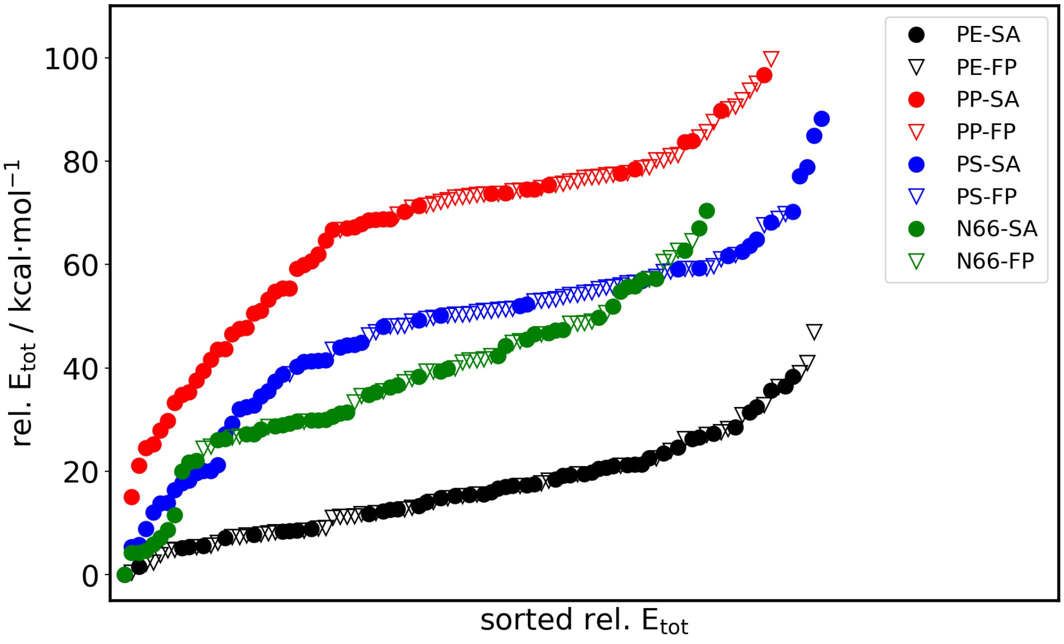

Figure S5. Total energy referenced to the global minimum value in kcal·mol^{-1}. Circles show the simulated annealing approach while open triangles show the FP approach. Note that the reference energy is the lowest energy of all structures from SA and FP together and all the data from both approaches are sorted together by increasing total energy.

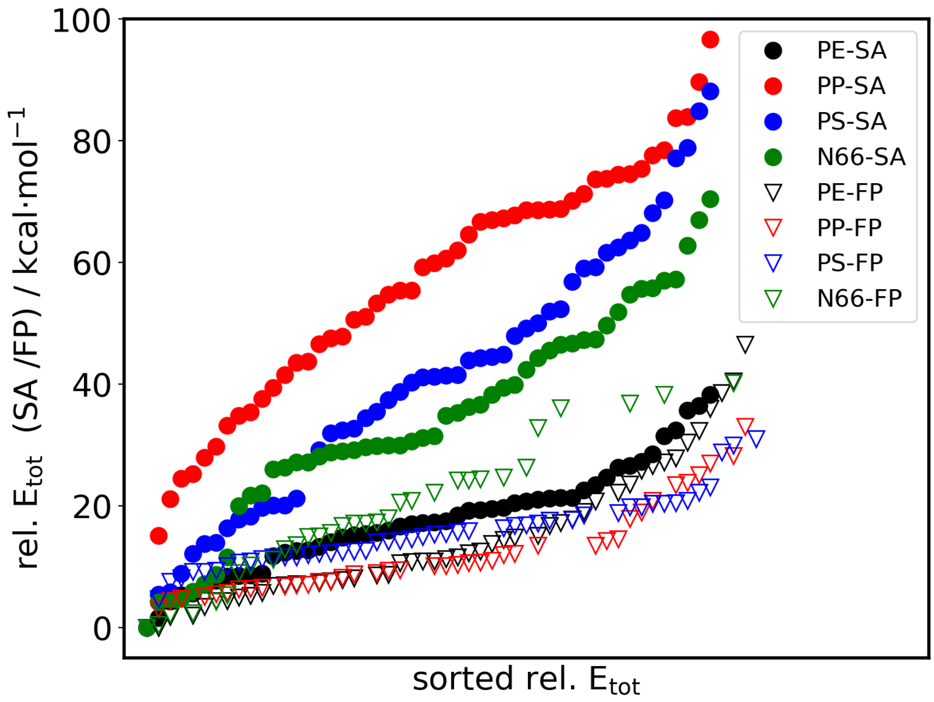

Figure S6. Total energy referenced to the respective minimum value in kcal·mol^{-1}. Circles show the simulated annealing approach while open triangles show the FP approach. Note that the reference energy is the lowest energy of all structures of the particular approach and the data for a given approach is sorted by increasing total energy.

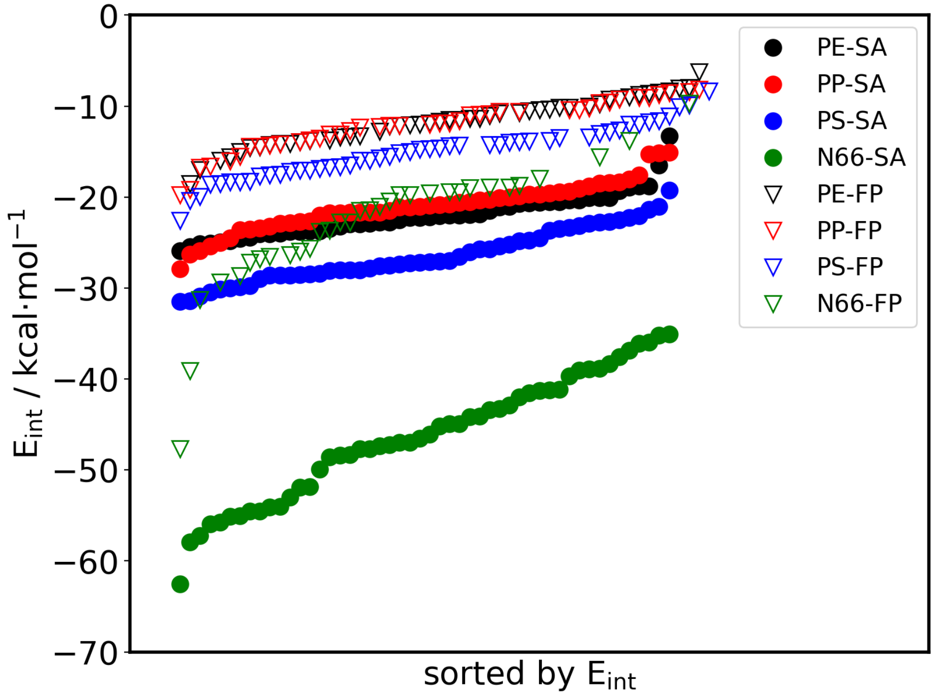

Figure S7. Interaction energy for the different TC@NP complexes in kcal·mol^{-1}. Circles show the simulated annealing approach while open triangles show the FP approach. Note that the data for a given approach is sorted by increasing interaction energy.

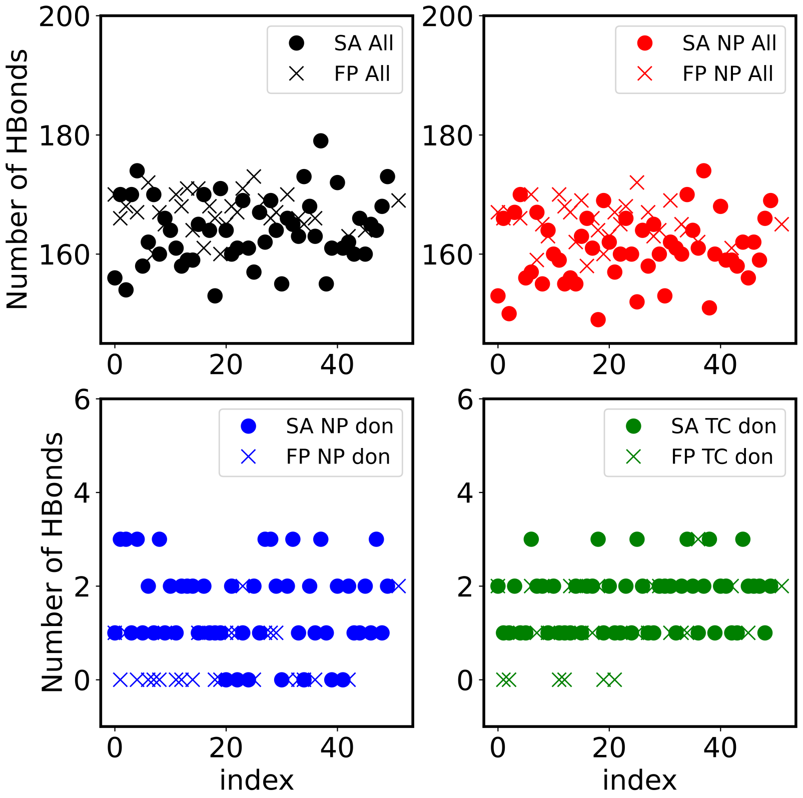

Figure S8. Analysis of hydrogen bonds in the TC@N66 systems. Upper left (black): All hydrogen bonds in the system. Upper right (red): All hydrogen bonds within the NP. Lower left (blue): All hydrogen bonds accepted by the TC molecules. Lower right (green): All hydrogen bonds donated by the TC molecule.

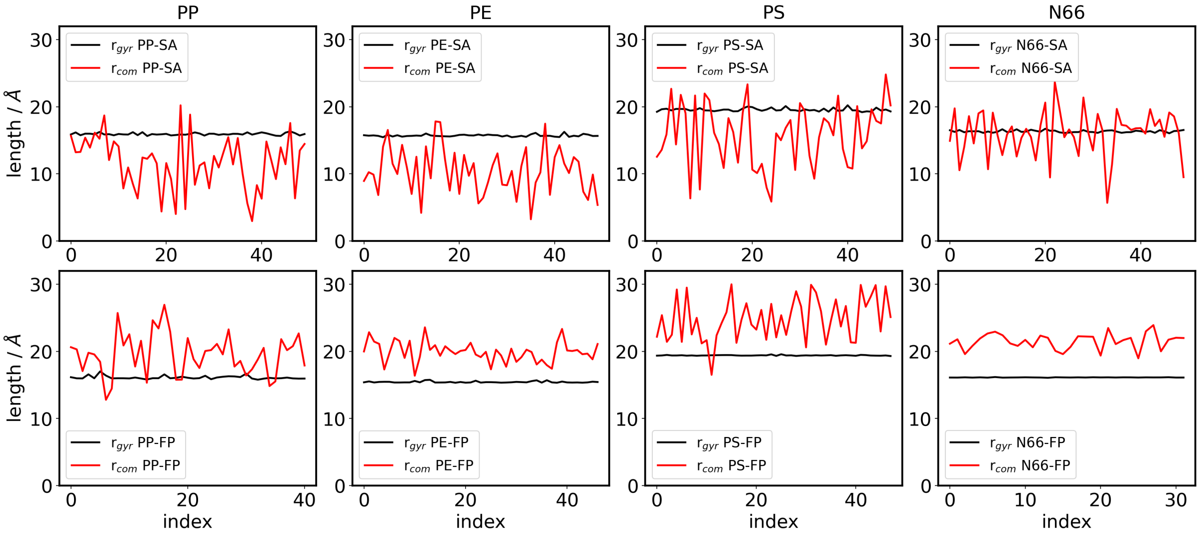

Figure S9. Radius of gyration and center of mass distances (TC–NP) versus index for all four TC@NP systems for SA (top) and FP (bottom).

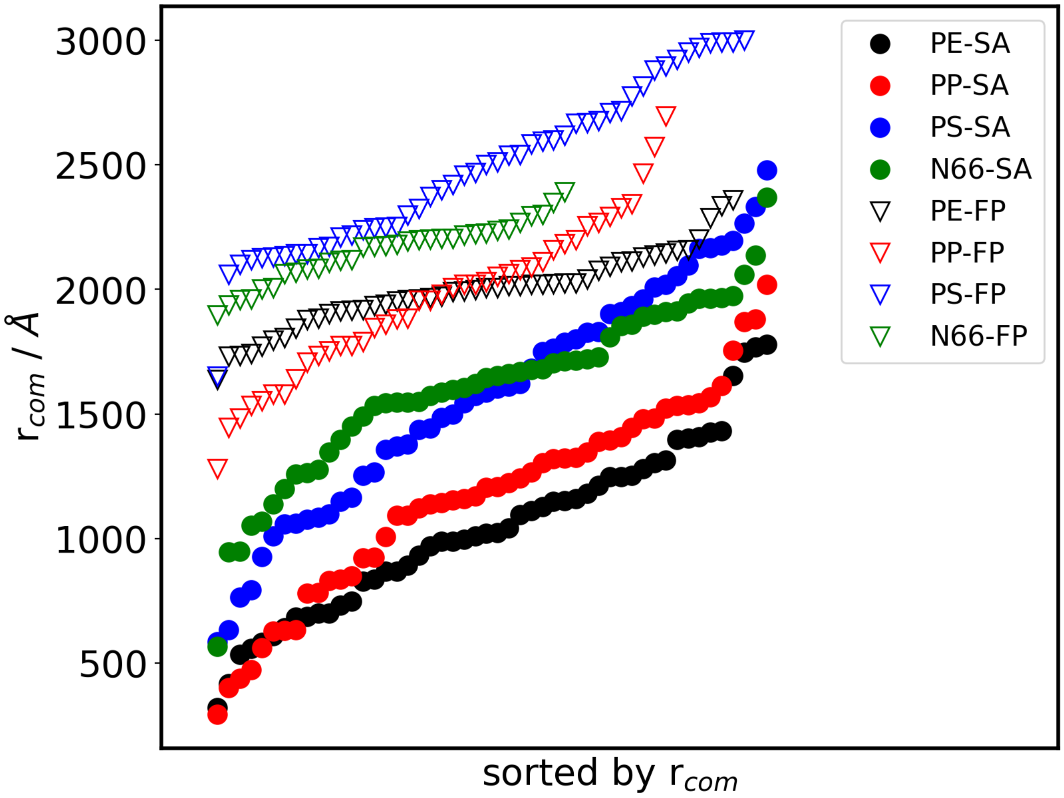

Figure S10. Center of mass distance (TC–NP) plotted against the index. Circles show the simulated annealing approach while open triangles show the FP approach. Note that the data for a given approach is sorted by increasing distance.

Table S3. Collection of mean, standard deviation (σ), 95 % confidence interval, skewness (skew), kurtosis (kurt), x-median (xMed), lowest x-value (xlow) and highest x-value (xhi) for distribution and bootstrapped distribution of the r_{com}/r_{gyr} ratio for all investigated TC@NP structures. SA: simulated annealing approach; SA^B: SA bootstrapped; FP: fixed particle approach; FP^B: fixed particle bootstrapped.

	PE				PP			
	SA	SA^B	FP	FP^B	SA	SA^B	FP	FP^B
mean	0.66	0.66	1.29	1.29	0.73	0.73	1.21	1.21
σ	0.22	0.03	0.10	0.01	0.25	0.04	0.19	0.03
95 % conf.	0.06	-	0.03	-	0.07	-	0.06	-
skew	0.19	−0.01	−0.06	0.01	−0.23	−0.03	0.04	0.01
kurt	−0.40	0.03	−0.02	0.02	−0.43	−0.01	−0.46	−0.03
xMed	0.65	0.66	1.29	1.29	0.76	0.73	1.22	1.22
xlow	0.20	0.54	1.05	1.24	0.19	0.60	0.78	1.10
xhi	1.14	0.78	1.51	1.34	1.27	0.87	1.63	1.32
	PS				N66			
	SA	SA^B	FP	FP^B	SA	SA^B	FP	FP^B
mean	0.80	0.80	1.28	1.28	0.98	0.98	1.33	1.33
σ	0.24	0.03	0.16	0.02	0.21	0.03	0.07	0.01
95 % conf.	0.07	-	0.05	-	0.06	-	0.03	-
skew	−0.21	−0.02	−0.04	−0.01	−0.68	−0.05	−0.32	−0.06
kurt	−0.78	−0.06	−0.65	0.01	0.58	−0.03	−0.47	0.06
xMed	0.81	0.80	1.28	1.28	1.01	0.98	1.35	1.33
xlow	0.29	0.69	0.85	1.19	0.34	0.87	1.18	1.28
xhi	1.27	0.92	1.54	1.37	1.44	1.09	1.48	1.38

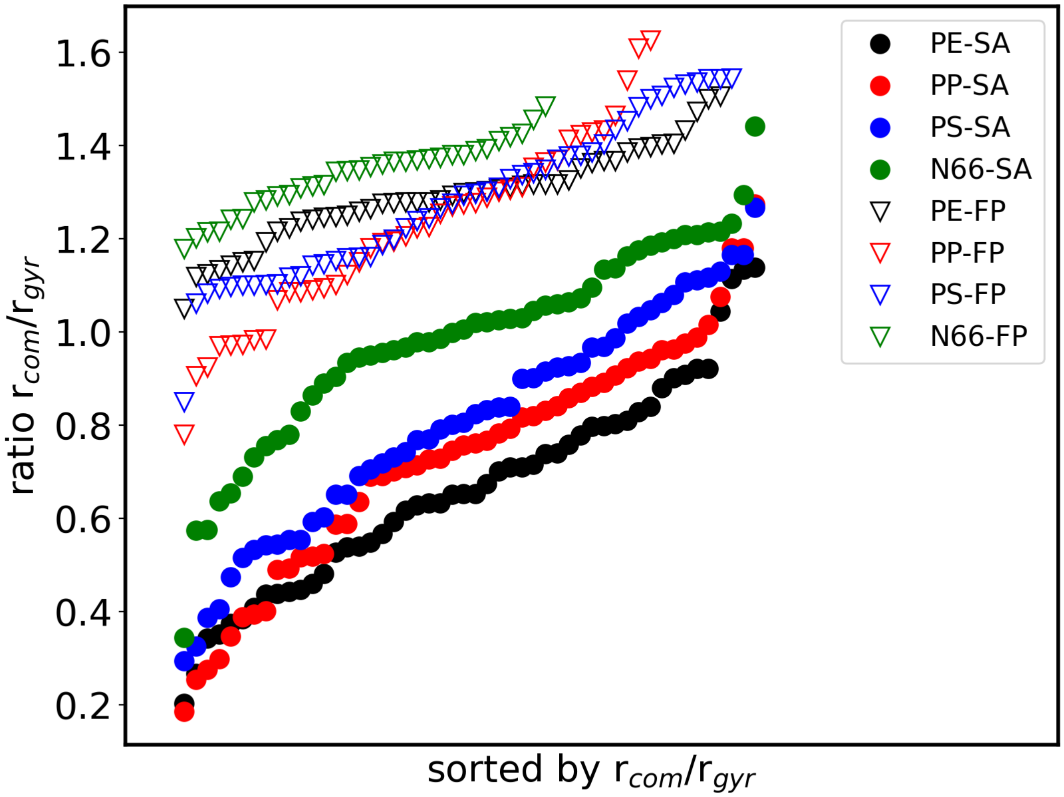

Figure S11. Ratio of the center of mass distance r_{com} (TC–NP) divided by the radius of gyration r_{gyr} of the NP. Circles show the simulated annealing approach while open triangles show the FP approach. Note that the data for a given approach is sorted by increasing ratios.

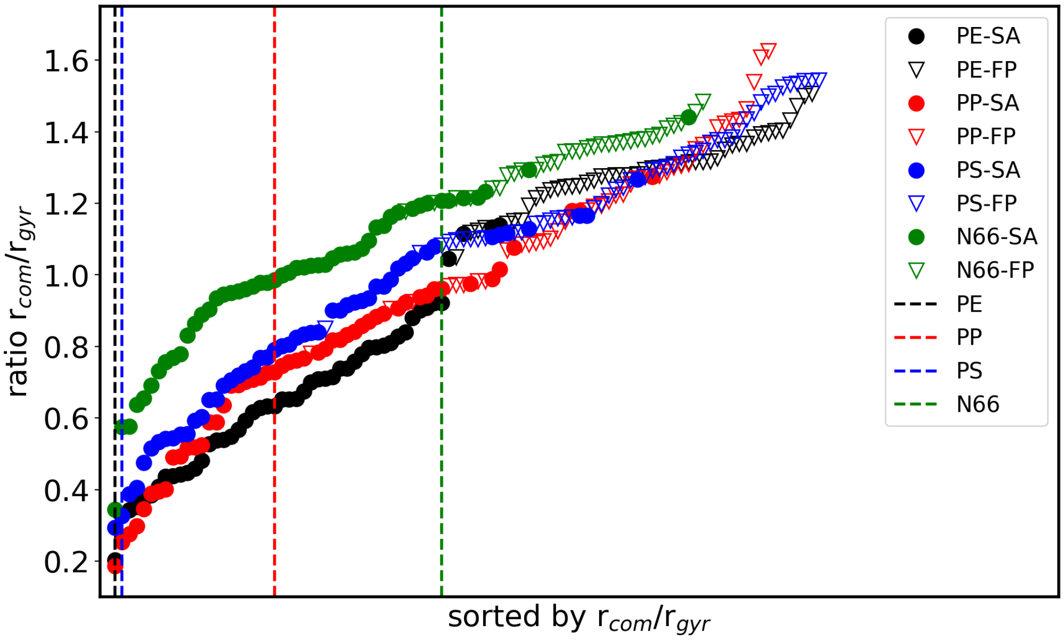

Figure S12. Ratio of the center of mass distance r_{com} (TC–NP) divided by the radius of gyration r_{gyr} of the NP. Circles show the simulated annealing approach while open triangles show the FP approach. The vertical lines mark the structures with the lowest total energies. Note that all the data from both approaches is sorted together by increasing ratio.

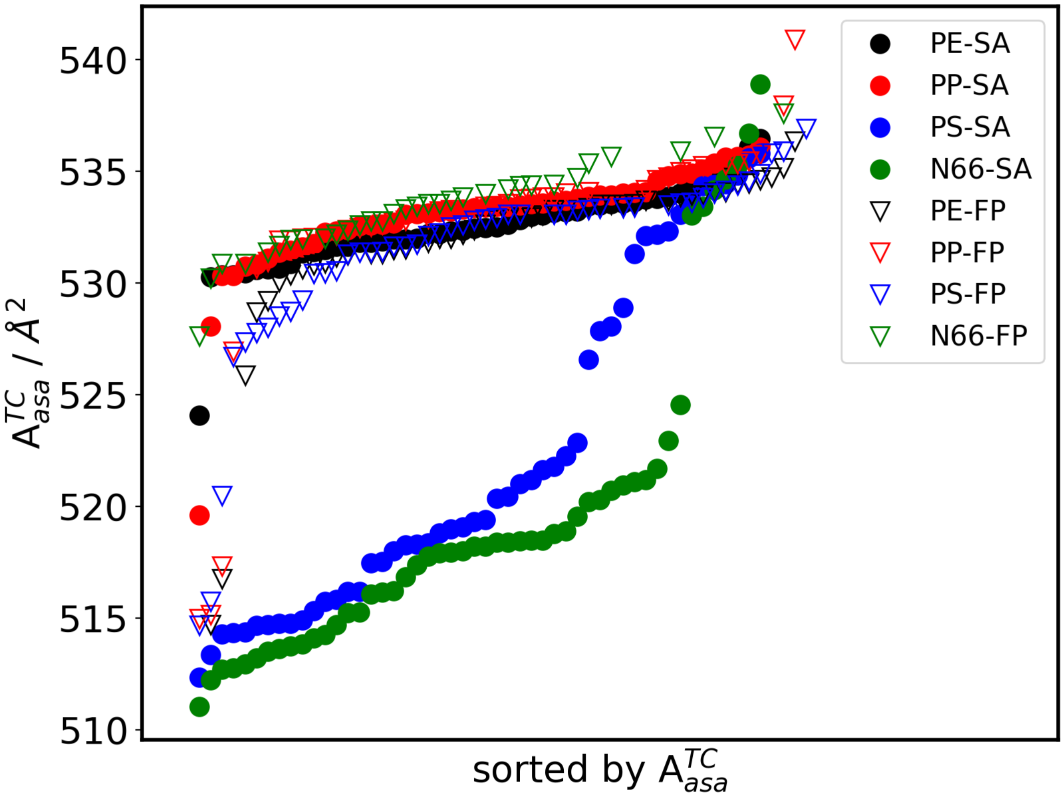

Figure S13. Accessible surface area of isolated TC molecules (A^{TC}_{asa}). Circles show the simulated annealing approach while open triangles show the FP approach. Note that the data for a given approach is sorted by increasing surface area.

Table S4. Collection of mean, standard deviation (σ), 95 % confidence interval, skewnesws (skew), kurtosis (kurt), x-median (xMed), lowest x-value (xlow) and highest xvalue (xhi) for distribution and bootstrapped distribution of the accessible surface area of the isolated TC molecules (A^{TC}_{asa}) for all investigated TC@NP structures. SA: simulated annealing approach; SA^B: SA bootstrapped; FP: fixed particle approach; FP^B: fixed particle bootstrapped.

	PE				PP			
	SA	SA^B	FP	FP^B	SA	SA^B	FP	FP^B
mean	532.5	532.5	532	532	532.9	532.9	532	532
σ	1.9	0.3	4	1	2.5	0.4	5	1
95 % conf.	0.6	-	1	-	0.7	-	2	-
skew	−1.31	−0.21	−3.10	−0.41	−3.18	−0.46	−2.36	−0.34
kurt	5.56	0.15	10.37	0.08	14.54	0.29	5.59	0.04
xMed	532.5	532.5	533	532	533.4	532.9	533	532
xlow	524.1	531.2	515	529	519.6	531.1	515	529
xhi	536.5	533.5	536	533	536.1	534.0	541	535
	PS				N66			
	SA	SA^B	FP	FP^B	SA	SA^B	FP	FP^B
mean	522	522	531	531	520	520	533.2	533.2
σ	7	1	4	1	7	1	1.9	0.3
95 % conf.	2	-	1	-	2	-	0.7	-
skew	0.60	0.06	−2.34	−0.32	1.40	0.20	−0.29	−0.06
kurt	−1.12	−0.01	5.88	0.05	1.05	0.00	0.82	0.08
xMed	519	522	533	531	518	520	533.4	533.2
xlow	512	518	515	529	511	516	527.6	531.8
xhi	536	526	537	533	539	524	537.6	534.5

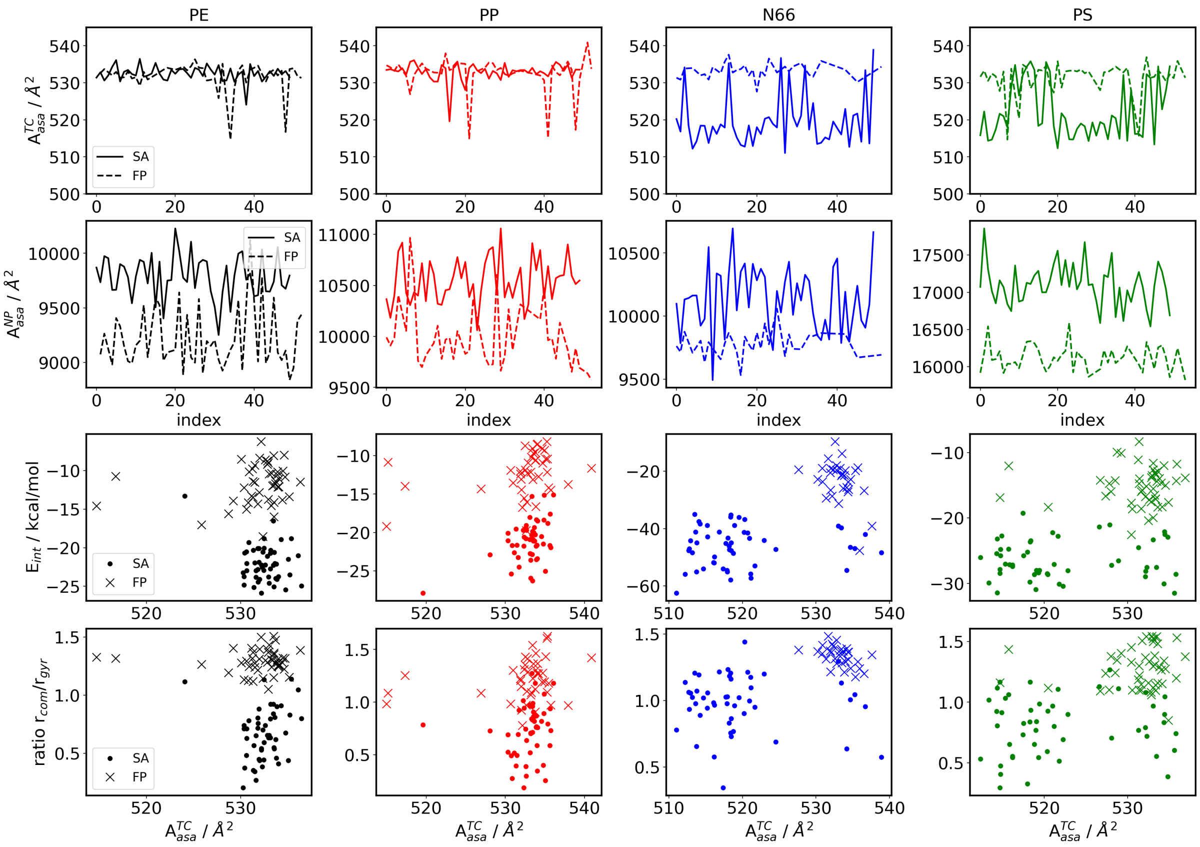

Figure S14. Accessible surface area plotted for all investigated TC@NP structures for the isolated TC (A^{TC}_{asa}; top row) and the isolated NP (A^{NP}_{asa}; second row). Interaction energies (E_{int}; third row) and r_{com}/r_{gyr} ratio (bottom row) plotted against the accessible surface area of isolated TC molecules A^{TC}_{asa} for all TC@NP structures.

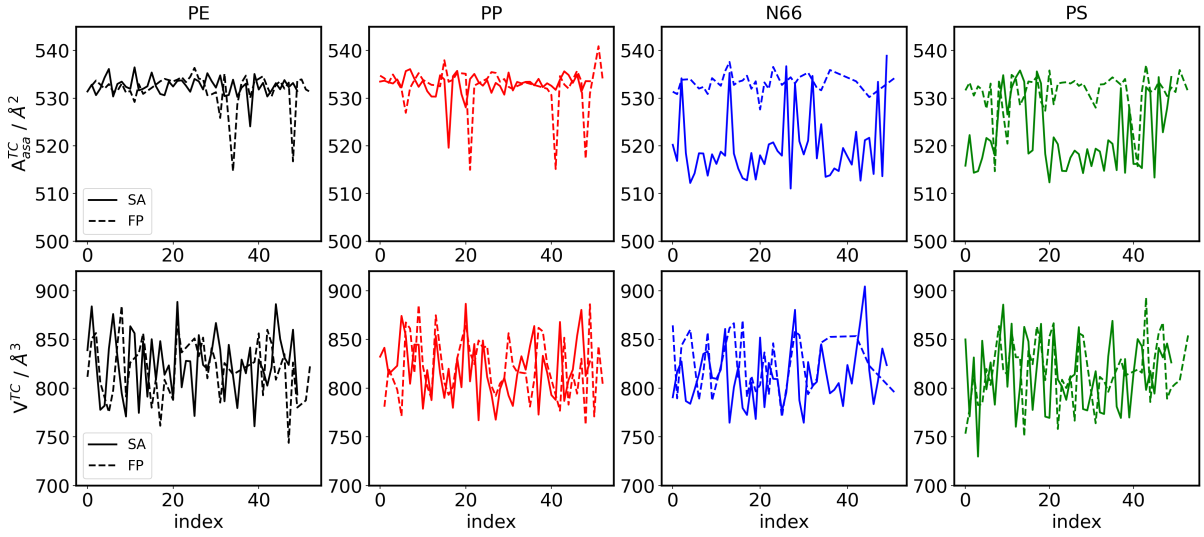

Figure S15. Accessible surface area (A^{TC}_{asa}; top) and respective volume (V^{TC}; bottom) of isolated TC molecules plotted for all investigated TC@NP structures.

Description of structures

The normalized distance $r(\mathrm{NP}-\mathrm{TC})$ that helps characterizing the structural relation of the TC with the NP is given by:

$$r(\mathrm{NP}-\mathrm{TC}) = \frac{\vec{r}_{\mathrm{NP}}^{\mathrm{CoM}} - \vec{r}_{\mathrm{TC}}^{\mathrm{CoM}}}{\sqrt{\frac{1}{|M|}\sum_{i=1}^{|M|}(\vec{r}_i^{\mathrm{CoM}} - \vec{r}_{\mathrm{NP}}^{\mathrm{CoM}})^2}},$$

where M is the set of all molecules that are part of the nano particle. It is the distance between the center of mass of both the particle $\vec{r}_{\mathrm{NP}}^{\mathrm{CoM}}$ and the drug $\vec{r}_{\mathrm{TC}}^{\mathrm{CoM}}$ divided by the molecule wise radius of gyration.

The atom wise weighted formulation of the radius of gyration results in:

$$r(\mathrm{NP}-\mathrm{TC}) = \frac{\vec{r}_{\mathrm{NP}}^{\mathrm{CoM}} - \vec{r}_{\mathrm{TC}}^{\mathrm{CoM}}}{\sqrt{\frac{1}{m_{\mathrm{NP}}}\sum_{i=1}^{N} m_i \cdot (\vec{r}_i - \vec{r}_{\mathrm{NP}}^{\mathrm{CoM}})^2}},$$

where N denotes the total number of atoms in the nano particle, m_i are the atomic masses of the respective atoms and m_{NP} the total mass of the nano particle.

This ratio can be understood as a normalization of the center of mass distance of the nanoparticle with the tetracycline, where unity means that the antibiotic sits exactly at the surface of the sphere with radius $r(\mathrm{NP}-\mathrm{TC})$ and center $r_{\mathrm{NP}}^{\mathrm{CoM}}$. This does not mean that it sits on the surface of the plastic particle, since the actual radius of a particle is not equal to it's radius of gyration. Especially for particles that are far from spherical (i.e. discs or rods), this ratio could be much lower than one even though the observed molecule is not penetrating the plastic particle. However, the general trend that a lower ratio $r(\mathrm{NP}-\mathrm{TC})$ corresponds to closer proximity or even more penetration of the tetracycline into the plastic particle still holds.

MD trajectory starting with short and large ratio

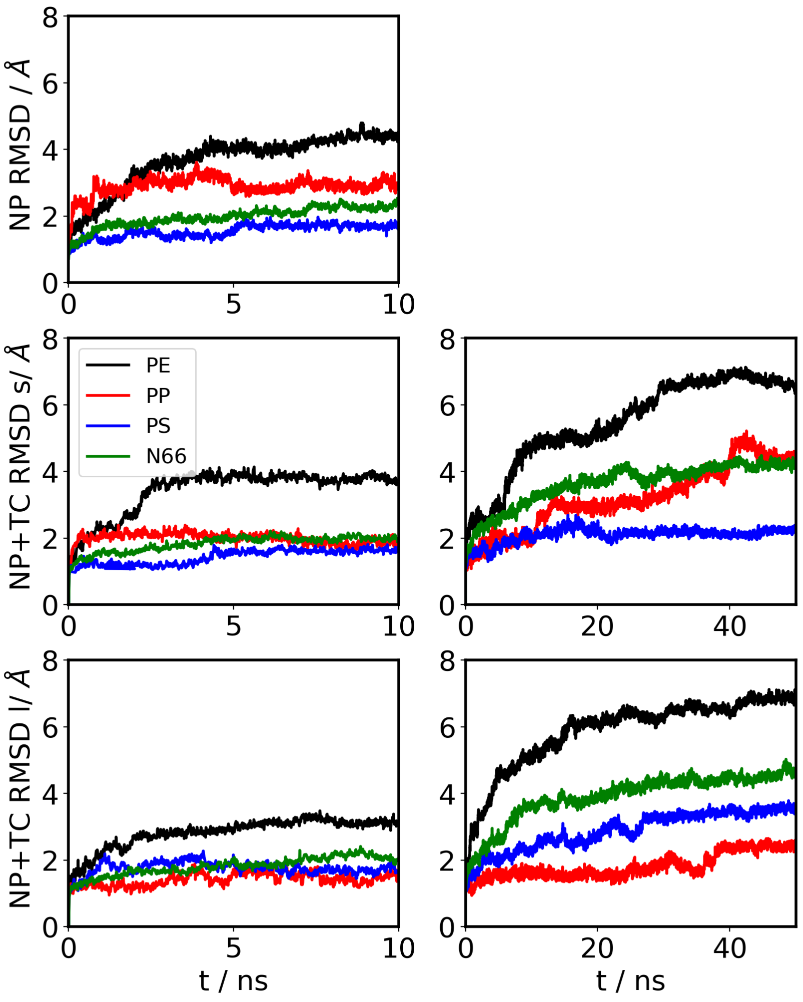

Figure S16. Root mean square deviation for the NP (top) and root mean square deviation of TC@NP complex (middle: initial structure with small r_{com}/r_{gyr} ratio; bottom: initial structure with large ratio). Left: last 10 ns; right: all 50 ns. Every time step was used for the analysis.

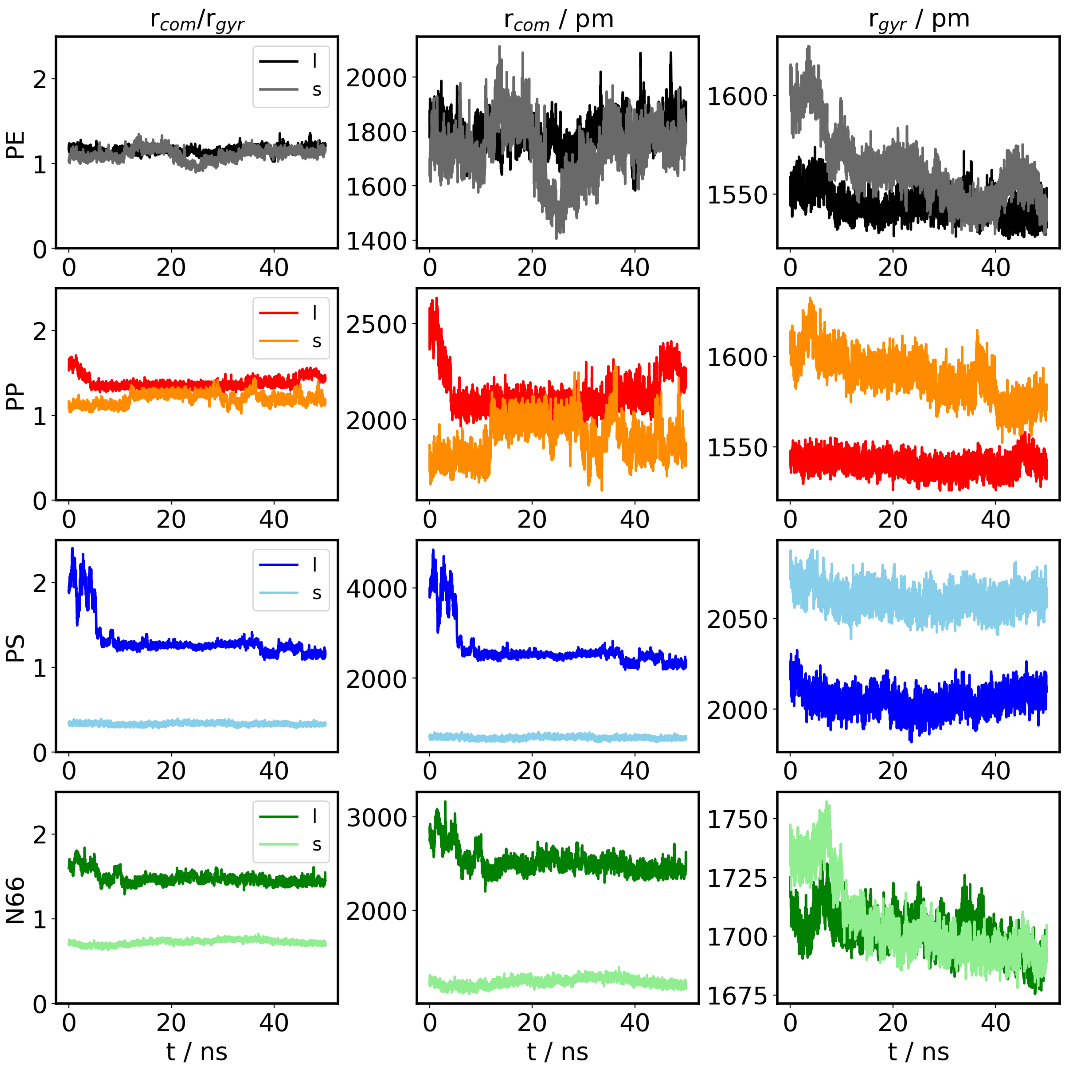

Figure S17. Center of mass distance (TC–NP) (middle), radius of gyration of NP (right) and ratio of the two (left) plotted against the simulation time for all investigated nanoplastics. Every tenth time step was used for the analysis.

Table S5. Average volume (V_{avg}) and average isoperimetric quotient (Q_{avg}) together with the respective standard deviations (σ_V; σ_Q) of all NP for the 50 ns simulations. Every tenth time step was used for the analysis.

	small ratio				large ratio			
Plastic	V_{avg}	σ_V	Q_{avg}	σ_Q	V_{avg}	σ_V	Q_{avg}	σ_Q
PE	32569	230	0.537	0.012	32534	248	0.542	0.012
PP	32051	234	0.491	0.012	32000	215	0.528	0.010
PS	60631	292	0.340	0.006	60587	294	0.356	0.010
N66	36900	182	0.308	0.009	36946	181	0.337	0.011

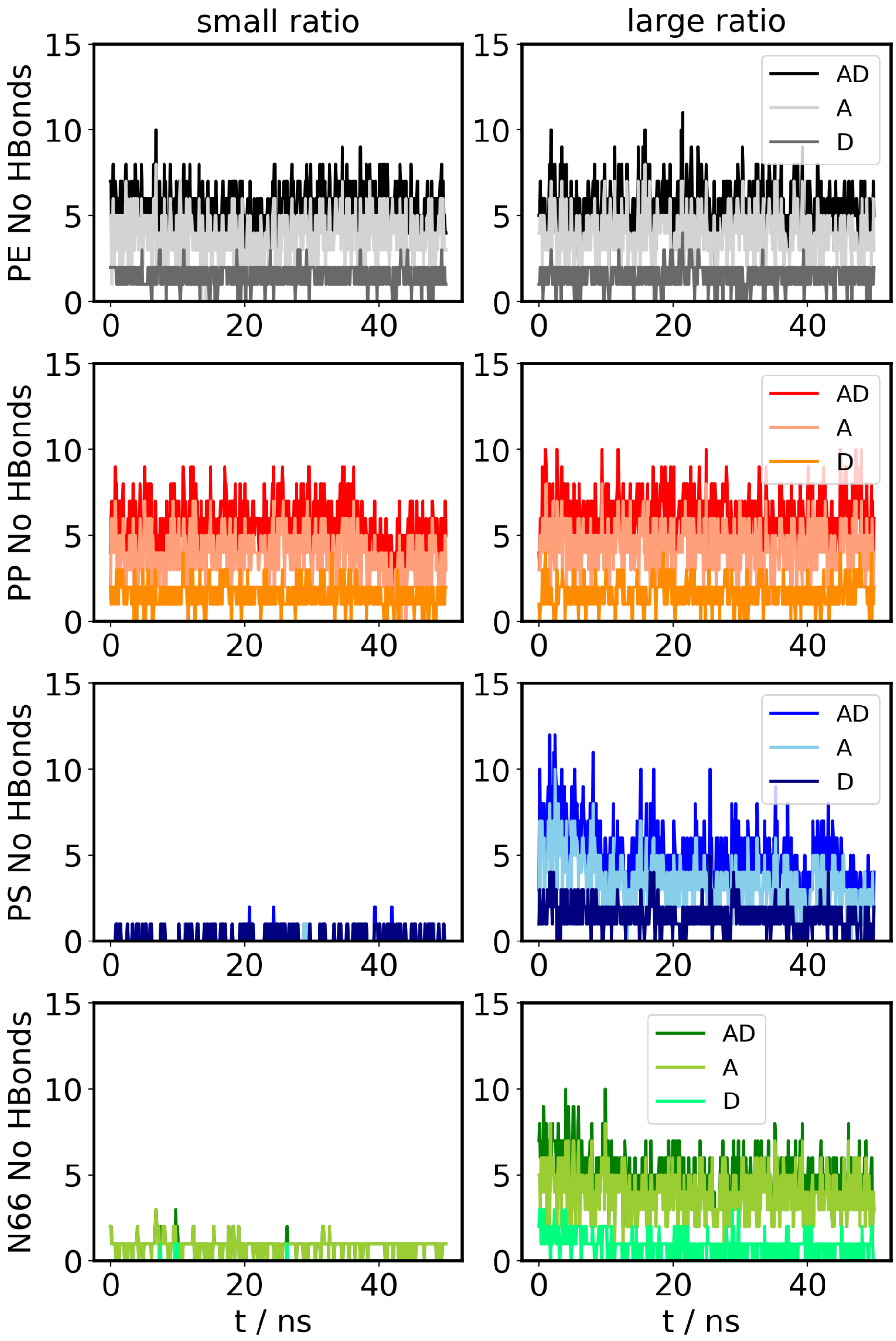

Figure S18. Number of hydrogen bonds plotted against the simulation time. AD/A/D indicates acceptor (A) and donor (D) hydrogen bonds from the viewpoint of the TC molecule to water. Left: trajectory starting from the small r_{com}/r_{gyr} ratio; Right: trajectory starting from the large ratio. Every tenth time step was used for the analysis.

Experimental section

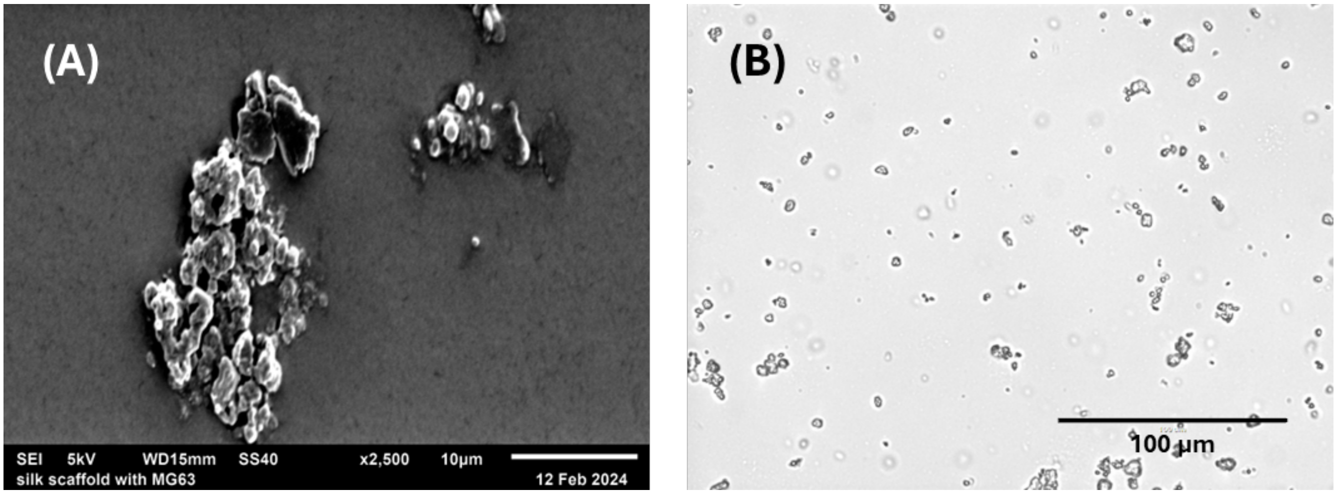

Figure S19. Microscopic analysis of in-lab produced PET particles. PET particles were produced in accordance with published procedures and analysed by (A) scanning electron microscopy and (B) light microscopy.

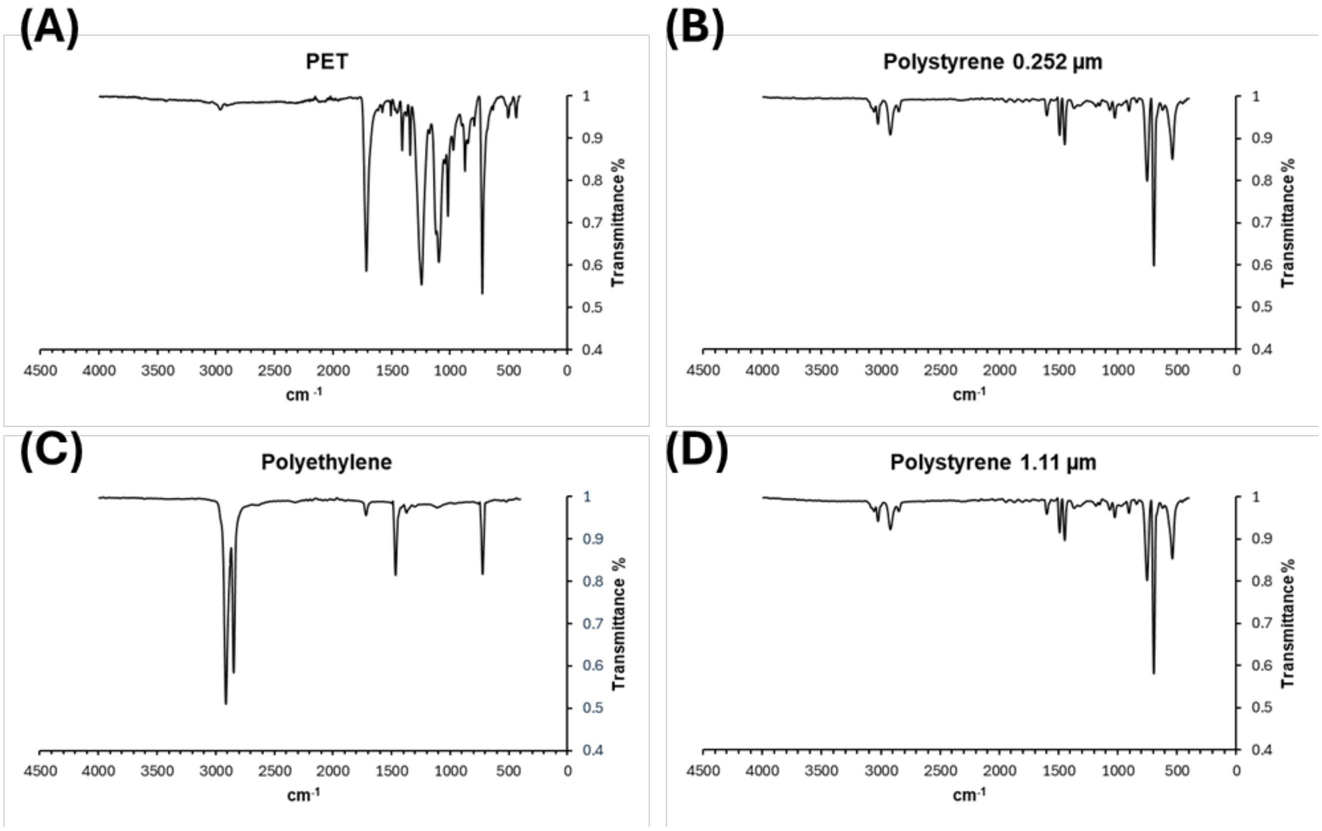

Figure S20. FTIR spectra of micro- and nanoplastic particles. As reference, FTIR spectra were recorded for (A) PET, (B) and (D) PS, and (C) PE particles.
