# Supplementary figures and images for: The adsorption of drugs on nanoplastics has severe biological impact
Source: Sci Rep. 2024 Oct 28;14:25853. doi: 10.1038/s41598-024-75785-4 (PMC11519658; doi:10.1038/s41598-024-75785-4)

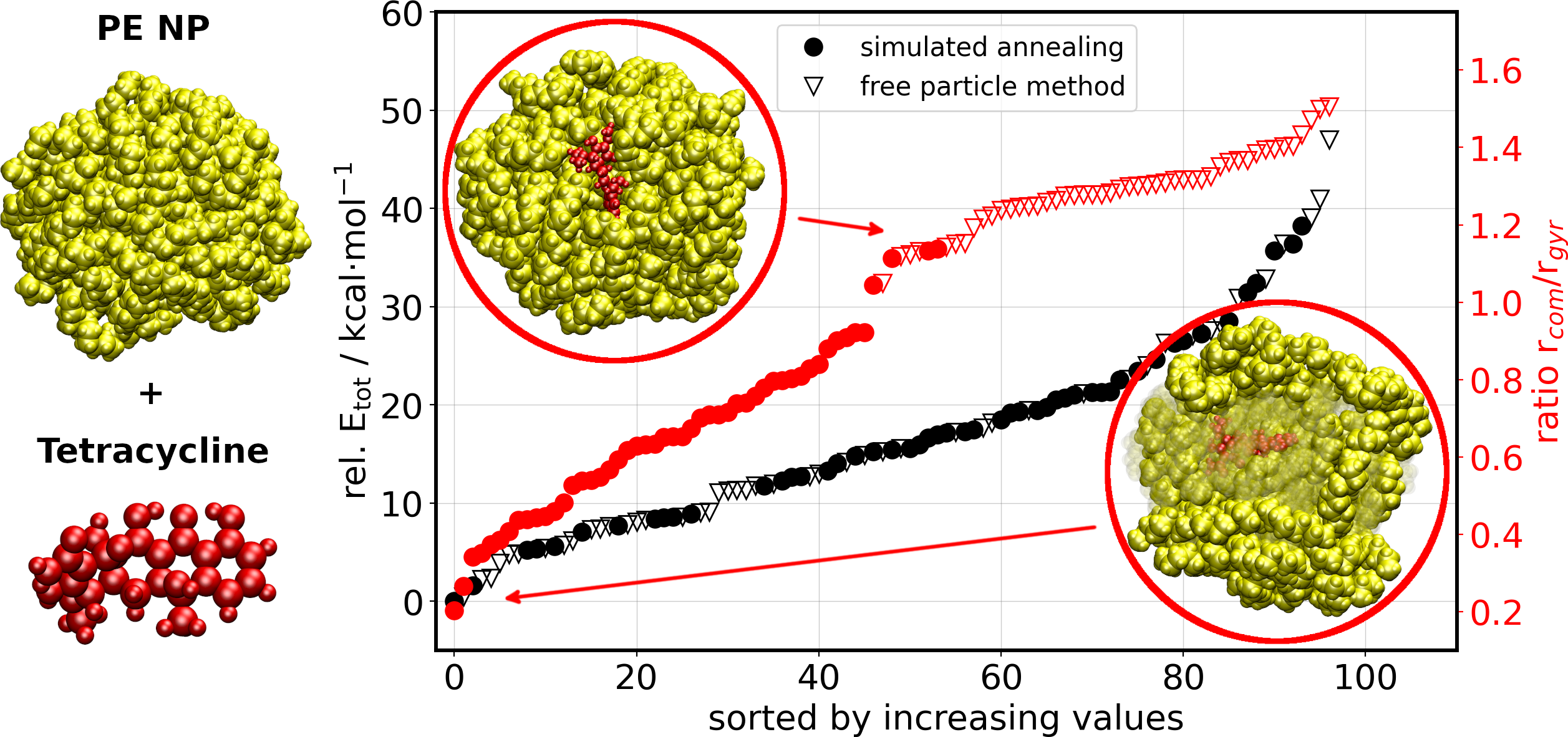

Supplement: Supplementary file 2 — Supplementary Information 2. [file 41598_2024_75785_MOESM2_ESM.png]
